# Anti-CMV IgG titer determines organ-specific protection toward immune checkpoint blockade-induced toxicities

**DOI:** 10.1136/jitc-2026-016050

**Published:** 2026-09-07

**Authors:** Gusztav Milotay, Martin Little, Sophie MacKay, Dylan Patrick Browne Muldoon, An-Nhi Huynh, Andrea Farkas, Shawn Sun, Guangyi Niu, Orion Tong, Chelsea A Taylor, Robert Watson, Harry Potts, Mark R Middleton, Paul Klenerman, Benjamin Fairfax

**Affiliations:** 1 Department of Oncology, University of Oxford, Oxford, UK; 2 MRC Weatherall Institute of Molecular Medicine, University of Oxford, Oxford, UK; 3 Oxford University Hospitals NHS Trust, Oxford, UK; 4 Nuffield Department of Medicine, University of Oxford, Oxford, UK; 5 Oncology, Oxford University Hospitals NHS Trust, Oxford, UK; 6 Translational Gastroenterology Unit, University of Oxford, Oxford, UK

**Keywords:** Immune related adverse event - irAE, Immune Checkpoint Inhibitor, Antibody, Biomarker

## Abstract

**Background:**

Immune-related adverse events (irAEs) post-immune checkpoint blockade (ICB) are a leading cause of patient morbidity. Robust peripheral biomarkers of irAEs are required to improve patient stratification to existing treatment regimens, and these are currently lacking. Seropositivity for human cytomegalovirus (CMV) is associated with protection against severe (grade 3+) irAEs post-ICB; however, the impact of infection on systemic immunity is highly variable. Here, in a prospectively recruited pan-cancer ICB-treated cohort (n=472 patients), we investigate a novel relationship between the relative baseline titer of anti-CMV IgG antibody and organ-specific protection against irAEs.

**Methods:**

Peripheral blood samples were collected from 472 patients prior to and following one cycle of ICB. CMV serotyping was performed on plasma, while flow cytometry and single-cell RNA/V(D)J sequencing were performed on peripheral blood mononuclear cells. Bulk RNA-sequencing was performed on sorted CD8^+^ T cells. Serological and phenotyping data were integrated with long-term clinical follow-up of response and irAEs.

**Results:**

In CMV seropositive patients, whereas anti-CMV IgG antibody level demonstrates stability over years, high pretreatment titer is independently associated with reduced all-organ grade 3+ irAEs. This pan-organ association subdivides into organ-specific effects; protection against non-colitis irAEs being observed only in those with an above median titer of anti-CMV IgG antibody (P_High titer vs CMV_−=2.1×10^−4^), whereas CMV-related protection against colitis is unrelated to titer (P_Low titer_=0.0012, P_High titer_=0.0031). We demonstrate that anti-CMV IgG antibody titer is robustly coupled to peripheral immune subset composition, with higher anti-CMV IgG titer associated with elevated CD4^+^ and CD8^+^ T cell cytotoxicity and effector cell expansion. Conversely, CMV seropositivity is associated with generally reduced circulating T_regs_ cells irrespective of titer. Furthermore, we find exacerbated T cell receptor repertoire skewing toward the largest clones in High Titer individuals, with reduced survival of these clones following ICB treatment.

**Conclusions:**

This work reinforces the importance of CMV in modulating ICB-induced irAEs, revealing a complex relationship between the degree of humoral anti-CMV immunity and organ-specific protection, while further highlighting the clinical utility of CMV serology in predicting ICB-induced irAEs.

**WHAT IS ALREADY KNOWN ON THIS TOPIC**

- Cytomegalovirus (CMV) infection is pervasive across populations and is a leading determinant of interindividual variation in systemic immunity. In recipients of immune checkpoint blockade (ICB) for melanoma, there appears to be a reduced incidence of grade 3+ immune-related adverse events (irAEs) in CMV-infected patients. There is significant variation in the magnitude of the anti-CMV immunoglobulin response across infected individuals, but how this relates to clinical outcomes, including protection against grade 3+ irAEs is unexplored.

**WHAT THIS STUDY ADDS**

- We investigated whether the magnitude of the anti-CMV humoral response provides additional clinically relevant information regarding the development of severe (grade 3+) irAEs following ICB. Using the Oxford Cancer Immunotherapy Toxicity and Efficacy cohort of prospectively recruited patients receiving ICB for cancer, we find that relative anti-CMV IgG titer shows distinct associations with T cell immunity. Clinically, grade 3+ colitis, the most common severe toxicity observed in our study, demonstrates reduced incidence irrespective of titer in CMV^+^ patients. Conversely, only patients with above median anti-CMV IgG titer demonstrated pan-organ protection against irAEs. This indicates a previously unrecognized relationship between the degree of humoral response to CMV and relative irAE protection.

**HOW THIS STUDY MIGHT AFFECT RESEARCH, PRACTICE OR POLICY**

- CMV serotyping forms a rapid, affordable, and non-invasive biomarker with potential translational utility. Tailored approaches to manage colitis in CMV− vs CMV+ patients may be warranted in ICB recipients, whereas such a strategy for severe non-colitis irAEs may only be indicated in those with high anti-CMV titers.

## Introduction

 Human cytomegalovirus (CMV) is a betaherpesvirus that causes lifelong latent infection in healthy individuals. While over 80% of people worldwide are estimated to be chronically infected with CMV, seroprevalence varies by country, being markedly lower in Australia,^1^ Western Europe,^2 3^ and the UK, where 56% of white participants in the UK Biobank were seropositive.^4^ It is increasingly clear that CMV has complex associations with cancer that may depend on cancer type and therapy.^5^ In assessment of patients with melanoma within the Oxford Cancer Immunotherapy Toxicity and Efficacy (OxCITE) study, a large, prospectively recruited pan-cancer dataset, we recently reported that IgG seropositivity for CMV is associated with improved overall survival post anti-PD-1 immune checkpoint blockade (ICB).^6^ Intriguingly, we also observed that CMV^+^ patients have a reduced risk of developing severe (grade 3+) immune-related adverse events (irAEs). Although people can be classified by CMV serostatus, among seropositive individuals there is marked variation in titers of anti-CMV IgG levels, potentially reflecting variation in humoral response to CMV. It is postulated that titer may relate to the degree of immune control of CMV,^7^ although in murine CMV infection, antibody titer is related to the size of the primary inoculate.^8^

Here, we extend our previous observations regarding CMV serostatus and irAEs by examining the relationship between anti-CMV IgG titer and irAE development in patients receiving ICB across multiple cancer types with samples from the OxCITE study. By increasing the sample size from the primary analysis (n=308 melanoma patients) to 472 patients across cancer types, as well as validating our original observations regarding CMV and ICB-related irAEs in internal discovery and validation cohorts (n=250 and n=222 patients, respectively), we explore the relationship between anti-CMV antibody titer and irAE development across all patients in OxCITE. We find that anti-CMV antibody titer provides additional information regarding organ-specific irAE risk and is linked to peripheral immune cell subset composition. Thus, in addition to serostatus, anti-CMV antibody titer may serve as a further irAE risk stratification biomarker of clinical utility.

## Results

### Anti-CMV titer is stable and independent of covariates

We assessed the clinical and immunological correlates of CMV IgG titer in two pan-cancer cohorts from the OxCITE study (n=250 and n=222, matched according to age, sex, CMV serostatus, and treatment) receiving either anti-PD-1 alone (sICB) or in combination with anti-CTLA-4 (cICB) as part of standard-of-care treatment. The cohorts consisted primarily of melanoma patients (n=382) as well as non-melanoma cancer subtypes treated by ICB in the absence of chemotherapy or other treatments (n=90) (tables 1–3).

**Table 1:** Cohort matching table

Characteristic	Discovery N=250*	Validation N=222*	P value†
Age	68 (56, 75)	68 (58, 76)	0.7
Sex			>0.9
Female	103 (41%)	91 (41%)	
Male	147 (59%)	131 (59%)	
Cancer			0.4
Melanoma	199 (80%)	183 (82%)	
Non-melanoma	51 (20%)	39 (18%)	
Treatment			>0.9
Adjuvant	35 (14%)	30 (14%)	
cICB	154 (62%)	138 (62%)	
sICB	61 (24%)	54 (24%)	
Titer			>0.9
CMV−	134 (54%)	120 (54%)	
Low titer	59 (24%)	50 (23%)	
High titer	57 (23%)	52 (23%)	

* Median (Q1, Q3); n (%).

† Wilcoxon rank sum test; Pearson’s χ2 test.

cICB, combination immune checkpoint blockade; CMV, cytomegalovirus; sICB, single ICB.

**Table 2:** Discovery cohort matching table for grade 3+ irAE development

Characteristic	Developed N=104*	Not developed N=146*	P value†
Age	66 (54, 73)	70 (57, 78)	0.014
Sex			0.4
Female	46 (45%)	57 (55%)	
Male	58 (39%)	89 (61%)	
Cancer			<0.001
Melanoma	97 (49%)	102 (51%)	
Non-melanoma	7 (14%)	44 (86%)	
Treatment			<0.001
Adjuvant	10 (29%)	25 (71%)	
cICB	79 (51%)	75 (49%)	
sICB	15 (25%)	46 (75%)	

* Median (Q1, Q3); n (%).

† Wilcoxon rank sum test; Pearson’s χ2 test.

cICB, combination immune checkpoint blockade; irAE, immune-related adverse event; sICB, single ICB.

**Table 3:** Validation cohort matching table for grade 3+ irAE development

Characteristic	Developed N=85*	Not developed N=137*	P value†
Age	65 (55, 74)	70 (60, 77)	0.014
Sex			0.3
Female	31 (34%)	60 (66%)	
Male	54 (41%)	77 (59%)	
Cancer			0.2
Melanoma	74 (40%)	109 (60%)	
Non-melanoma	11 (28%)	28 (72%)	
Treatment			<0.001
Adjuvant	2 (6.7%)	28 (93%)	
cICB	73 (53%)	65 (47%)	
sICB	10 (19%)	44 (81%)	

* Median (Q1, Q3); n (%).

† Wilcoxon rank sum test; Pearson’s χ2 test.

cICB, combination immune checkpoint blockade; irAE, immune-related adverse event; sICB, single ICB.

**Figure 1:**
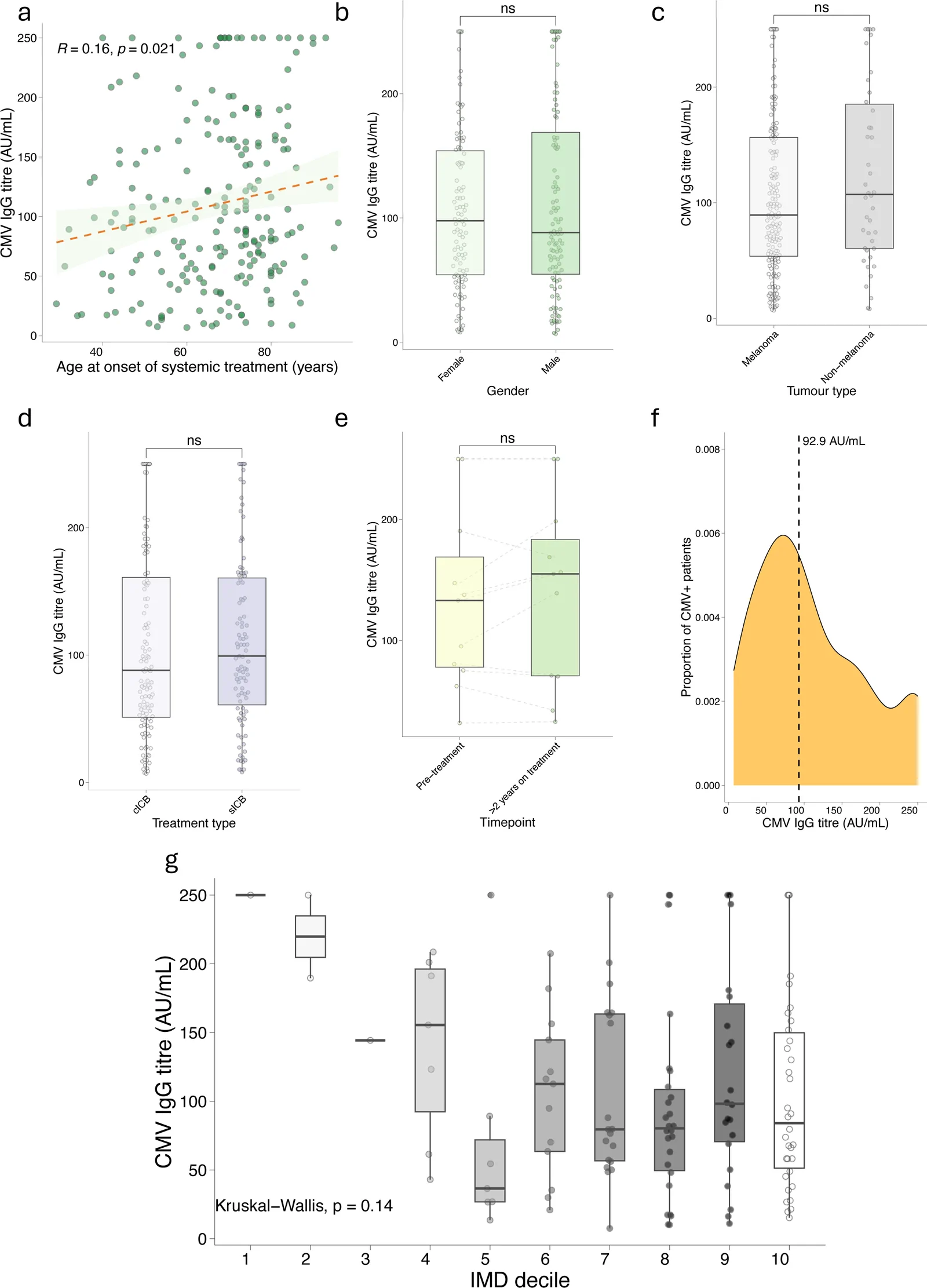
Clinical correlates of anti-CMV IgG titer. (**a**) Correlation of anti-CMV IgG titer (a.u.mL^−1^) and age at onset of ICB treatment (n=218, Spearman’s rank correlation test, R denotes the Spearman’s r and p value is derived from a two-sided t-test). (**b**) Anti-CMV IgG titer (a.u.mL^−1^) according to biological sex (n_Male_=112, n_Female_=106, p=0.81). P values result from the application of a Wilcoxon rank sum test; lower and upper box hinges represent the 25th to 75th percentiles, the central line represents the median, and whiskers extend to the highest and lowest values no greater than 1.5×IQR. (**c**) Anti-CMV IgG titer (a.u.mL^−1^) according to tumor type (n_Melanoma_=176, n_Non-melanoma_=42, p=0.23, Wilcoxon rank sum test, boxplot as in (b)). (**d**) Anti-CMV IgG titer (a.u.mL−1) according to treatment regimen (n_cICB_=119, n_sICB_=99, p=0.49, Wilcoxon rank sum test, boxplot as in (b)). (**e**) Anti-CMV IgG titer (a.u.mL^−1^) at pretreatment and late timepoints (*>*2 years on treatment) (n=11 patients, p=0.48, Wilcoxon signed-rank test, boxplot as in (b)). (**f**) Distribution of anti-CMV IgG titers (a.u.mL^−1^) in seropositive patients (n=218) with median titer (92.9 a.u.mL^−1^ indicated). (**g**) Comparing anti-CMV IgG titers (a.u.mL^−1^) across Index of Multiple Deprivation (IMD) deciles in CMV seropositive patients (patients as in (f), Kruskal-Wallis test, boxplot as in (b)). CMV, cytomegalovirus; ICB, immune checkpoint blockade.

The relationship between pretreatment CMV IgG titer and clinical covariates was examined across both cohorts. In the CMV^+^ cohort (n=218), we observed a marginal relationship between titer and age at onset of systemic treatment (r=0.16, p=0.021), supporting a weak link between the titer of anti-CMV IgG and duration of infection (figure 1a).^9^ We did not observe associations between anti-CMV IgG titer and other covariates, including biological sex, tumor type, and treatment regimens (figure 1b–d). To further address whether anti-CMV IgG titer remains stable over time, we selected a cohort of CMV^+^ patients (n=11) who responded to ICB for at least 2 years and compared their post-treatment to pretreatment titers. A significant change in titer was not detected over time (p=0.48, Wilcoxon signed-rank test), reinforcing limited coupling between titer and time since primary infection (figure 1e), consistent with other longitudinal studies.^10 11^ Given CMV IgG titers were not normally distributed (figure 1f), the seropositive cohort was divided into high and low titer groups, based on the median value (92.9 a.u.mL^−1^, 185.8 a.u.mL^−1^ in undiluted plasma, see Methods), and such divisions were used for all downstream analysis (n=254 CMV^−^, n=109 CMV^+^_Low titer_, n=109 CMV^+^_High titer_). CMV infection has previously been shown to be associated with socioeconomic status; therefore, we also assessed whether CMV seropositivity was differentially distributed across Index of Multiple Deprivation (IMD) deciles, finding there was no significant difference in this patient cohort (p=0.55, χ^2^ test).^12 13^ Finally, we assessed whether the distribution of titers varied significantly according to IMD decile, again finding no significant difference (p=0.14, Kruskal-Wallis test, figure 1g).

### CMV titer alters organ specificity of severe irAEs

**Figure 2:**
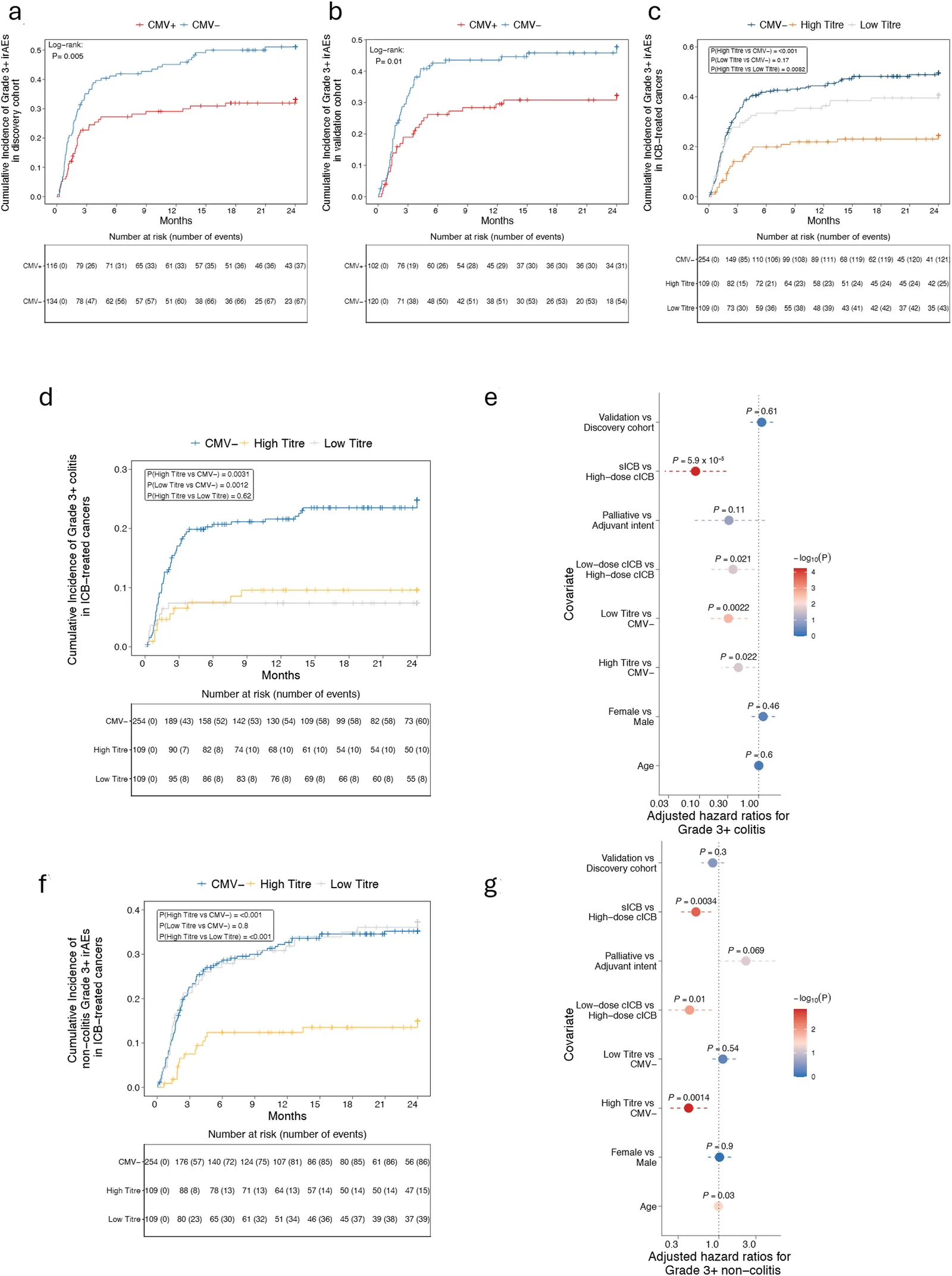
CMV titer is associated with diverse organ-specific risk of severe irAEs. (**a**) Cumulative incidence of developing grade 3+ irAEs across ICB-treated patients in discovery cohort is significantly reduced in CMV^+^ patients (p=0.005, nCMV+=116 and n_CMV_−=134, two-sided log-rank test). (**b**) Same analysis as a for validation cohort (p=0.01, nCMV+=102 and n_CMV_−=120, one-sided log-rank test). (**c**) Same analysis as a indicating that risk of grade 3+ irAEs is reduced in a CMV IgG titer-dependent fashion (n_CMV_−=254, n_Low titer_=109, n_High titer_=109, two-sided log-rank test). (**d**) Semicompeting risks analysis of grade 3+ colitis suggests that both CMV^+^_High titer_ and CMV^+^_Low titer_ patients have reduced risk compared with CMV^−^ patients (two-sided log-rank test). (**e**) Multivariable semicompeting risk analysis, where death is a semicompeting risk, highlights that both CMV^+^_High titer_ and CMV^+^_Low titer_ individuals are protected against severe colitis when adjusting for treatment regimen, tumor type, age, cohort, and sex (HR_adj_ High titer=0.45, P_High titer_=0.022; HR_adj_ Low titer=0.31, P_Low titer_=0.0022, Wald test; patients as in (a)) (**f**) Same analysis as d for non-colitis grade 3+ irAEs. (**g**) Same analysis as (c) for severe irAEs, excluding colitis, indicates that CMV^+^_High titer_ individuals are protected against these toxicities (HR_adj_=0.41, p=0.0014). CMV, cytomegalovirus; cICB, combination immune checkpoint blockade; irAEs, immune-related adverse events; sICB, single ICB.

**Figure 3:**
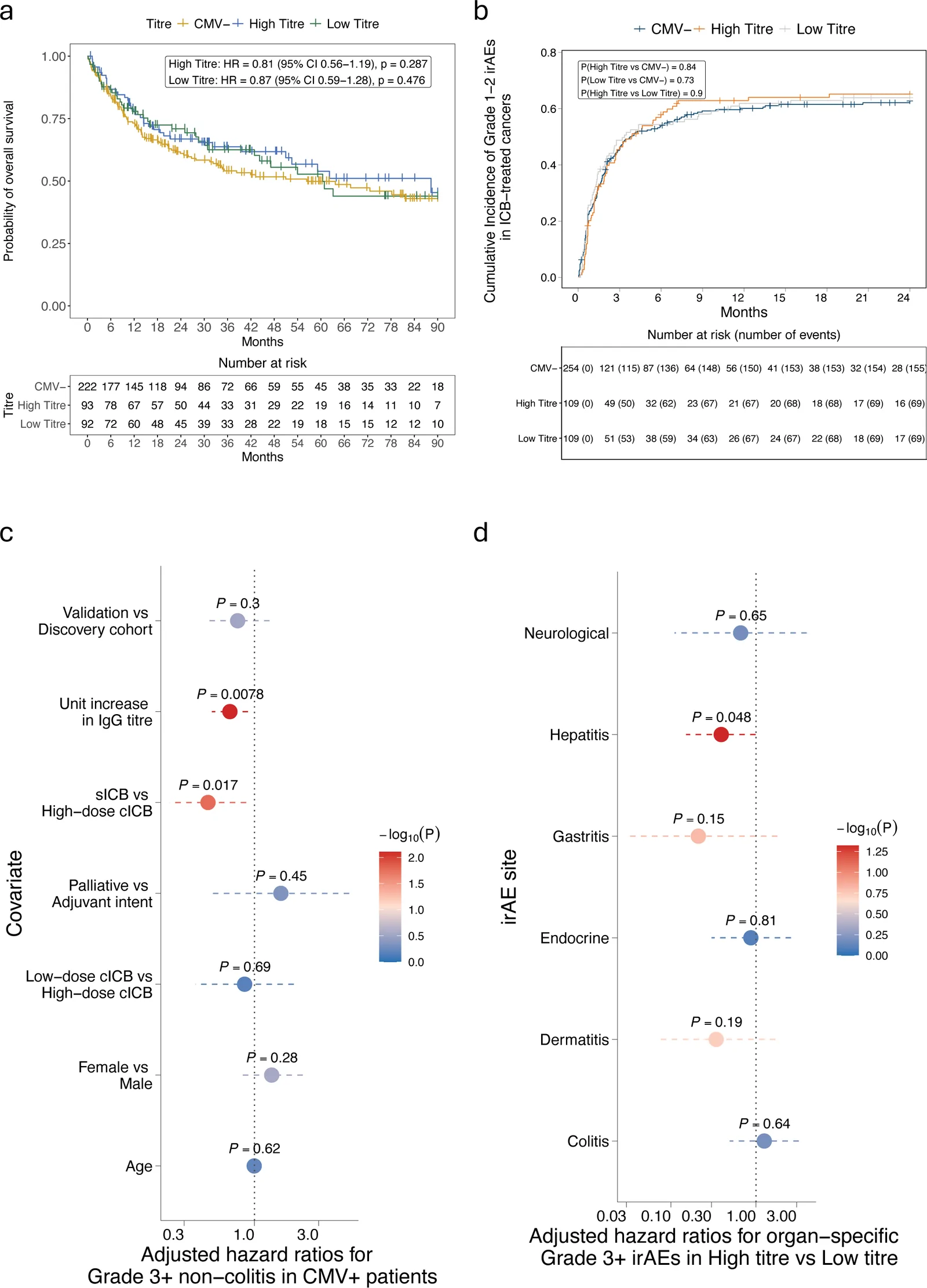
Organ-specific irAE risk within CMV^+^ patients according to anti-CMV IgG titer. (**a**) Kaplan-Meier analysis demonstrates no significant difference in overall survival according to CMV titer in metastatic cancers (n=407). (**b**) Cumulative incidence of grades 1–2 irAEs according to CMV titer (n_CMV_−=254, n_Low titer_=109, n_High titer_=109, two-sided log-rank test). (**c**) Multivariable semicompeting risk analysis, where death is a semicompeting risk, highlights that a scaled and centered anti-CMV IgG titer predicts risk of grade 3+ non-colitis irAEs when adjusting for treatment regimen, tumor type, age, cohort, and sex. (**d**) Univariate semicompeting risks assessing organ-specific irAE development across CMV+High titer versus CMV+Low titer patients. cICB, combination immune checkpoint blockade; CMV, cytomegalovirus; irAE, immune-related adverse event; sICB, single ICB.

Revisiting our previous association between CMV seropositivity and reduced risk of grade 3+ irAEs in melanoma, we extended our observations, dividing this larger pan-cancer dataset into discovery (n=250) and internal validation (n=222) cohorts. For this analysis, we used a semicompeting risks model which accepts non-terminal events (irAEs) and terminal events (death), which may preclude the non-terminal events from occurring, while treating terminal events as informative censoring rather than dropout events. This method allowed us to assess the risk of toxicity over time, accounting for the fact that some individuals may die prior to irAE development. Using this approach, we found CMV seropositivity was associated with significantly reduced risk of all grade 3+ irAEs across both cohorts (P_Discovery_=0.005 and P_Validation_=0.01) (figure 2a,b). To be sufficiently powered to assess the influence of IgG titer on severe toxicities, we performed a univariate semicompeting risks analysis where death was a competing risk^14^ on the combined cohort (n=472). All downstream analysis was performed using both cohorts unless stated otherwise. Using this approach, we found that CMV^+^_High titer_ patients were significantly less likely to develop grade 3+ irAEs compared with CMV^+^_Low titer_ (p=0.0082) and CMV seronegative (p=2.7×10^−5^) counterparts (figure 2c). Conversely, across all irAEs, CMV^+^_Low titer_ patients did not show a significant reduction in grade 3+ irAEs compared with CMV seronegative patients, although this smaller sample size greatly reduced power and a trend toward protection was observed (p=0.17). Nonetheless, this analysis demonstrates that, in addition to CMV serostatus, the titer of anti-CMV antibody forms a further core determinant of irAE development. Given the relationship between CMV titer and irAE development, we investigated the impact of IgG titer on overall survival in metastatic cancers (n=407) but found no significant difference compared with CMV^-^ patients, reinforcing the decoupling of high-grade irAEs from oncological response to ICB^15^ (figure 3a). Furthermore, as per our previous observation of absence of CMV-related protection against low-grade irAEs,^6^ we found no influence of anti-CMV IgG titer on low-grade irAEs, again highlighting that CMV appears to most impact development of high-grade irAEs (figure 3b).

We previously noted that CMV protection against irAEs showed organ-specificity, with protection against colitis, the most common severe side effect observed in the OxCITE study, being strongest (OR vs CMV^−^=0.39). Notably, in this current analysis, we find that CMV protection against colitis is independent of anti-CMV IgG titer, with both CMV^+^_High titer_ and CMV^+^_Low titer_ groups demonstrating reduced risk of grade 3+ colitis compared with CMV^−^ patients (p=0.0031 and p=0.0012, respectively), while the effect of titer was non-significant (p=0.62), indicative of pan-CMV protection in the gut (figure 2d). A similar result was observed in a multivariable analysis controlling for age, biological sex, cohort, ICB regimen, and treatment intent, with both CMV^+^_High titer_ and CMV^+^_Low titer_ patients being protected against colitis compared with CMV^−^ patients (figure 2e, P_High titer_=0.022, P_Low titer_=0.0022). The other significant covariate associated with colitis was ICB regimen, with high-dose cICB (3 mg:1 mg kg^−1^ of ipilimumab:nivolumab for metastatic melanoma, see online supplemental material) demonstrating the known increased risk of ipilimumab with irAEs.^16^

Given the discrepancy between grade 3+ irAEs and colitis, we next investigated the risk of non-colitis grade 3+ toxicities. Strikingly, we found that, contrary to colitis, only CMV^+^_High titer_ patients demonstrated reduced cumulative incidence of grade 3+ irAEs in other organs compared with both CMV^+^_Low titer_ and CMV seronegative patients (p=2.1×10^−4^ and p=3.4×10^−4^, respectively) (figure 2f). Meanwhile, no difference was observed between CMV^+^_Low titer_ and CMV seronegative patients (p=0.8). Again, this observation was confirmed using multivariable analysis where CMV^+^_High titer_ individuals were protected against non-colitis grade 3+ irAEs (p=0.0014, figure 2g), whereas no effect was noted for the CMV^+^_Low titer_ group (p=0.54). To assess whether the difference within the CMV^+^ patients was continuous across titers, we assessed the risk of developing non-colitis grade 3+irAEs within the CMV^+^ patients using IgG titer as a scaled and centered predictive variable. Accounting for clinical covariates, we found decreased risk with elevated titers existed along a continuum (p=0.0078, figure 3c). Next, we explored, using a time-independent and time-dependent sensitivity analysis, for the most informative threshold of anti-CMV IgG titer for the occurrence of non-colitis grade 3+ irAEs within a 24-month window of commencing ICB, identifying the cut-off as 95.2a.u./mL^−1^ (190.4a.u./mL^−1^ in undiluted plasma). Given this was similar to the median of 92.9a.u./mL^−1^, we proceeded to use this median value in all downstream analyses. Finally, we assessed whether the signal for non-colitis was driven by any other major organ-specific irAEs, finding that alone, only grade 3+ hepatitis was moderately decreased in CMV^+^_High titer_ individuals relative to their CMV^+^_Low titer_ counterparts (p=0.048, figure 3d). We anticipate that this is likely due to the reduced power to detect differences between individual organs where grade 3+ irAEs are rarer (tables 4 and 5).

**Table 4:** Discovery cohort breakdown of organ-specific grade 3+ irAEs according to CMV titer

Characteristic	CMV−	Low titer	High titer	Events in all patients
	N=91*	N=91*	N=12*	
Cardiac	2 (40%)	3 (60%)	0 (0%)	5/250
Colitis	32 (82%)	3 (7.7%)	4 (10%)	39/250
Dermatitis	4 (44%)	4 (44%)	1 (11%)	9/250
Endocrine	9 (53%)	6 (35%)	2 (12%)	17/250
Gastritis	3 (60%)	2 (40%)	0 (0%)	5/250
Hepatitis	17 (57%)	9 (30%)	4 (13%)	30/250
Nephritis	2 (40%)	3 (60%)	0 (0%)	5/250
Neurological	3 (43%)	3 (43%)	1 (14%)	7/250
Other	5 (71%)	2 (29%)	0 (0%)	7/250
Pneumonitis	3 (75%)	1 (25%)	0 (0%)	4/250
Rheumatological	11 (69%)	5 (31%)	0 (0%)	16/250

* n (%).

CMV, cytomegalovirus; irAE, immune-related adverse event.

**Table 5:** Validation cohort breakdown of organ-specific grade 3+ irAEs according to CMV titer

Characteristic	CMV−	Low titer	High titer	Events in all patients
	N=80*	N=25*	N=15*	
Cardiac	0 (0%)	1 (100%)	0 (0%)	1/222
Colitis	28 (72%)	5 (13%)	6 (15%)	39/222
Dermatitis	1 (25%)	2 (50%)	1 (25%)	4/222
Endocrine	11 (69%)	1 (6.3%)	4 (25%)	16/222
Gastritis	5 (56%)	3 (33%)	1 (11%)	9/222
Hepatitis	14 (64%)	6 (27%)	2 (9.1%)	22/222
Nephritis	2 (40%)	3 (60%)	0 (0%)	5/222
Neurological	3 (75%)	0 (0%)	1 (25%)	4/222
Other	3 (75%)	1 (25%)	0 (0%)	4/222
Pneumonitis	5 (100%)	0 (0%)	0 (0%)	5/222
Rheumatological	8 (73%)	3 (27%)	0 (0%)	11/222

* n (%).

CMV, cytomegalovirus; irAE, immune-related adverse event.

### High CMV titer protects against severe irAEs across treatment types

**Figure 4:**
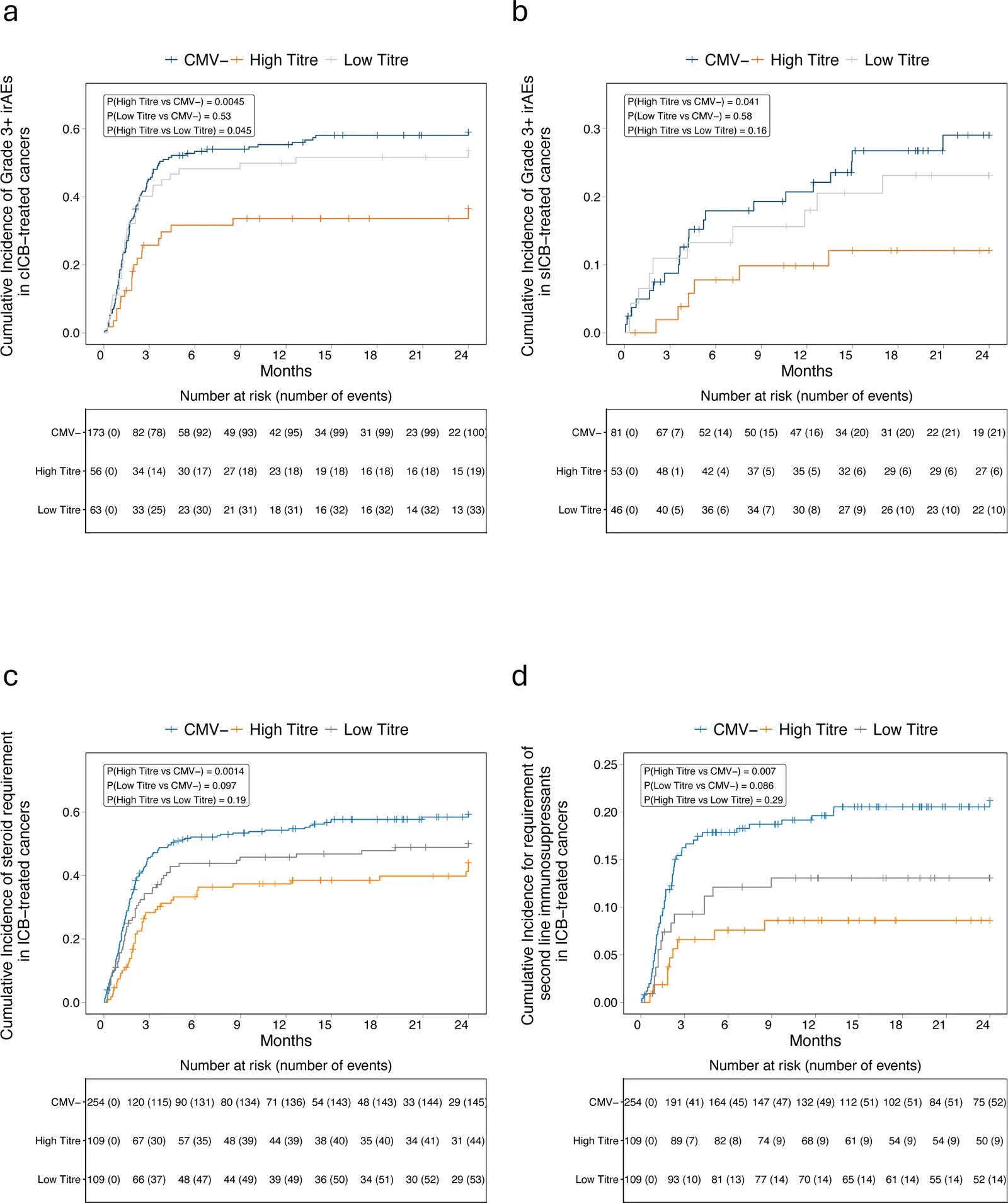
Grade 3+ irAEs according to treatment type and impact on toxicity management. (**a**) Cumulative incidence of developing grade 3+ irAEs in cICB-treated patients according to CMV titer (n_CMV_−=173, n_Low titer_=63, n_High titer_=56, two-sided log-rank test). (**b**) As in a for sICB-treated patients (n_CMV_−=81, n_Low titer_=46, n_High titer_=53), highlighting that CMV^+^_High titer_ individuals have reduced risk of grade 3+ irAEs relative to CMV− patients (p=0.041). Cumulative incidence of steroid requirement (**c**) and second-line immunosuppression (**d**) in ICB-treated patients according to CMV IgG titer (n_CMV_−=254, n_Low titer_=109, n_High titer_=109, two-sided log-rank test). cICB, combination immune checkpoint blockade; CMV, cytomegalovirus; irAEs, immune-related adverse events; sICB, single ICB.

Grade 3+ irAEs affect >60% of patients receiving cICB compared with just 10%–15% receiving sICB in trial and real-world data, highlighting that ipilimumab is the major driver of severe toxicities.^16–18^ There is evidence to support target-specific activity at the organ level, however.^19^ Given this, we sought to explore whether CMV^+^_High titer_ patients were protected from grade 3+ irAEs irrespective of treatment type. In the cICB cohort, we found that CMV^+^_High titer_ patients had a reduced cumulative incidence of severe toxicities compared with CMV seronegative individuals (p=0.0045, n_High titer_=56, n_CMV_− =173) and also compared with the CMV^+^_Low titer_ individuals (p=0.045, n_Low titer_=63), consistent with the complete cohort (figure 4a). Despite high-grade toxicities being rare following sICB treatment and fewer patients receiving this treatment, we still observed that CMV^+^_High titer_ sICB recipients had reduced risk compared with their CMV seronegative counterparts (p=0.041, n_High titer_=53, n_CMV_^_−_^=81), indicating that the interaction with anti-CMV IgG titer is not restricted to ipilimumab-induced adverse events (figure 4b).

### CMV serostatus is a stronger determinant of immunosuppressant requirements to manage irAEs compared with IgG titer

High-dose corticosteroids are used as treatment for irAEs, an effect that is increasingly recognized to abate the anti-cancer effects of ICB.^20 21^ To assess whether titer influenced steroid requirement following an irAE, we performed a semicompeting risks analysis between CMV IgG titer and steroid use, the competing risk being death. We found that, although CMV^+^_High titer_ individuals had reduced steroid requirement compared with CMV seronegative patients (p=0.0014), no significant difference was observed between CMV^+^_High titer_ and CMV^+^_Low titer_ patients (p=0.19) (figure 4c), although a trend toward increased protection in the former was observed. We also explored the use of second-line immunosuppressants given in the context of steroid failure to control irAEs, finding a significantly reduced requirement for second-line immunosuppression in CMV^+^ patients, although this showed a similar direction of effect across both the CMV^+^_High titer_ patients (P _High titer vs CMV_^_−_^=0.007) and CMV^+^_Low titer_ patients (P _Low titer vs CMV^−^_=0.086), with no discernible difference within CMV^+^ patients (P_High vs Low_=0.29) (figure 4d). In summary, this data suggests that irAE management is strongly associated with CMV serostatus, with CMV seropositive patients less likely to require both steroids and second-line immunosuppressants, with only a moderate effect of anti-CMV IgG titer. In part, this observation likely reflects the absence of association between anti-CMV IgG titer and development of colitis, which is the irAE most likely to require both steroids and second-line immunosuppression.

### Immunological correlates of CMV titer

**Figure 5:**
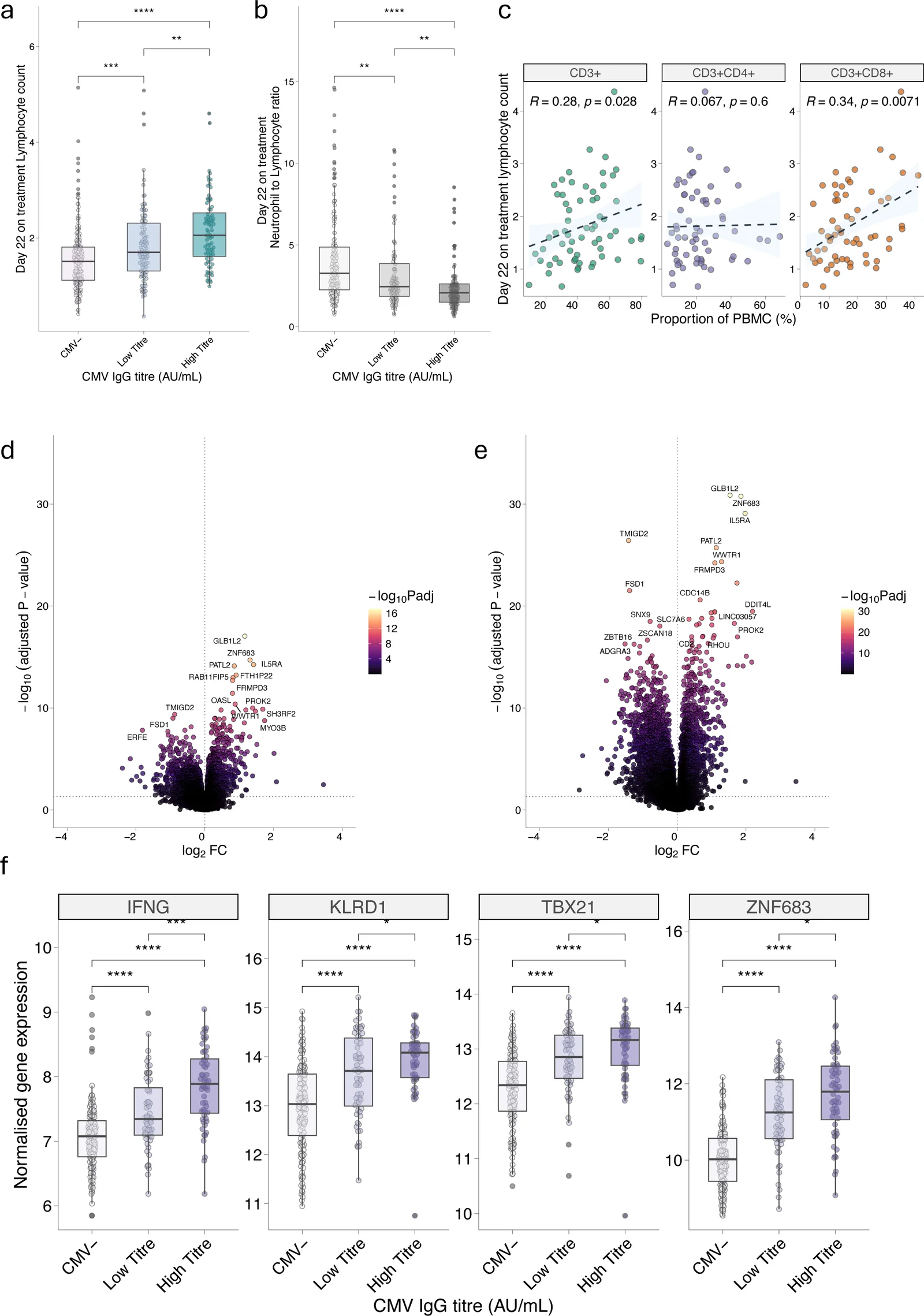
Immunological correlates of CMV titer. (**a**) On-treatment lymphocyte count is elevated in a titer-dependent manner; p values derived from Kruskal-Wallis and post hoc Dunn tests. Lower and upper box hinges represent the 25th to 75th percentiles, the central line represents the median, and whiskers extend to the highest and lowest values no greater than 1.5×IQR. (**b**) On-treatment neutrophil:lymphocyte ratio according to CMV IgG titer, p values and boxplots as in (a). (**c**) Correlation of on-treatment lymphocyte count and CD3^+^, CD3^+^CD4,^+^ and CD3^+^CD8^+^ T cells as a proportion of (PBMC) (n=63, Spearman’s rank correlation test, R denotes the Spearman r*,* and p value is derived from a two-sided t-test). (**d**) Differentially expressed genes (DEGs) between CMV^+^_Low titer_ and CMV^−^ patients from pretreatment CD8^+^ T cells; y-axis shows Benjamini-Hochberg-corrected-log_10_(Padj) derived from negative binomial Wald test using CMV^−^ samples as a reference (n_Low titer_=60, n_CMV_−=145). (**e**) As (c), assessing differential gene expression between CMV^+^_High titer_ and CMV− patients (n_High titer_=56, n_CMV_−=145). (**f**) Normalized *IFNG*, *KLRD1*, *TBX21,* and *ZNF683* expression increases in a dose-dependent manner from CMV^−^ to CMV^+^_Low titer_ to CMV^+^_High titer_ patients (n_CMV_−=145, n_Low titer_=60, n_High titer_=56; p values derived from Kruskal-Wallis and post hoc Dunn tests, boxplots as in (a)). CMV, cytomegalovirus; PBMC, peripheral blood mononuclear cell. *P* < 0.05, **P < 0.01, ***P < 0.001, ****P < 0.0001.

Given the divergent irAE profiles within the CMV^+^ cohort according to IgG titer, we sought to identify immunological correlates of CMV serology. First, we assessed hematological indices from routine blood tests, identifying CMV^+^ patients had higher on-treatment lymphocyte count in a titer-dependent fashion (n=361, P_High titer vs Low titer_=0.0077, P _Low titer vs CMV_^_−_^ = 7×10^−4^, figure 5a). As per our previous findings, we found that this resulted in a reduction in neutrophil:lymphocyte ratio in CMV^+^_High titer_ patients (n=328, P_High titer vs Low titer_=0.0052, figure 5b), which is a well-characterized negative prognostic biomarker.^6 22 23^ As the raised lymphocyte count following CMV infection is associated with inflation of the CD8^+^ T cell compartment specifically (figure 5c), we explored CD8^+^ T cell RNA-sequencing data from pretreatment CMV^+^_Low titer_ (n=60) and CMV^-^ patients (n=145), identifying 3324 differentially expressed genes (DEGs) (figure 5d, see online supplemental material).^24^ Interestingly, when comparing CMV^+^_High titer_ (n=56) with the same CMV^−^ cohort, we found a significantly larger set of 6773 DEGs (figure 5e, see online supplemental material), indicating that an elevated anti-CMV IgG titer is associated with magnified T cell compartment transcriptomic differences between CMV^+^ and CMV^-^ patients. Among the DEGs, we found key markers of cytotoxicity and tissue residency that demonstrated further induction in CMV^+^_High titer_ relative to CMV^+^_Low titer_ patients. Specifically, we determined the normalized expression of *IFNG*, a marker of cytotoxicity, *KLRD1*, involved in HLA-E recognition by NK and effector T cells, *TBX21*, a key transcription factor for type I T cell differentiation, and *ZNF683,* which is a driver of tissue residency.^25–28^ All these genes have been reported to play essential roles in shaping both anti-viral and anti-tumor responses.^25–28^ Across these markers, we found that CMV^+^_High titer_ patients had elevated expression relative to both CMV^+^_Low titer_ and CMV^−^ individuals, most notably for *IFNG* (P_High vs CMV^−^_=2.7×10^−14^, P_High vs Low_=1.9×10^−4^, figure 5f), suggesting further expansion of effector cells in the T cell compartment of these patients.

**Figure 6:**
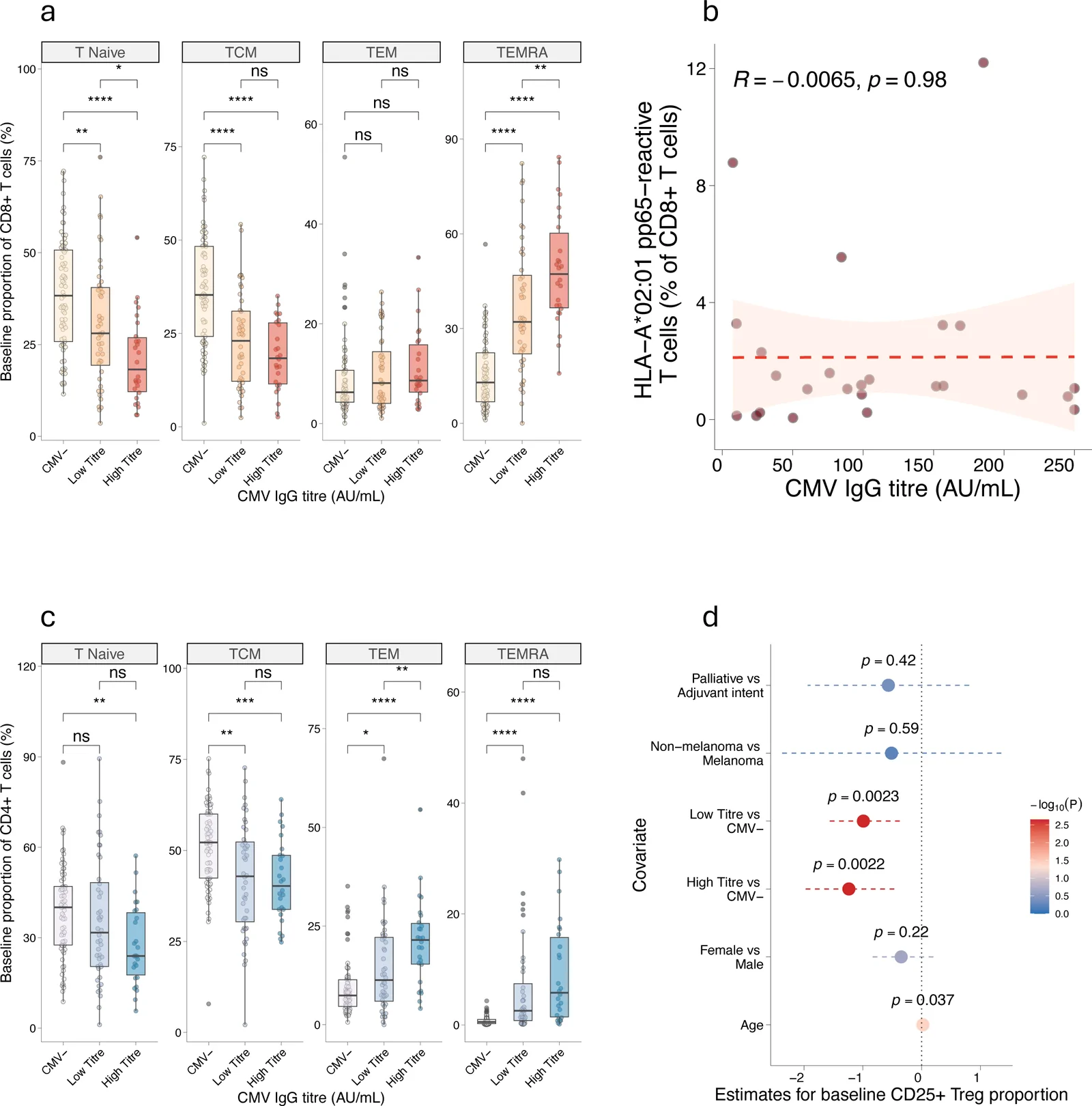
T cell subsets according to CMV titer. (**a**) Flow cytometry-derived pretreatment CD8^+^ T cell subsets flow cytometry according to CMV titer (n_CMV_−=73, n_Low titer_=44, n_High titer_=26, p values derived from Kruskal-Wallis and post hoc Dunn tests). Lower and upper box hinges represent the 25th to 75th percentiles, the central line represents the median, and whiskers extend to the highest and lowest values no greater than 1.5×IQR. (**b**) No association between CMV titer (a.u.mL^−1^) with HLA-A*02:01 pp65-reactive CD8^+^ T cells (n=25, r=−0.0065, p=0.98, Spearman’s rank correlation test, R denotes the Spearman r*,* and p value is derived from a two-sided t-test). (**c**) CD4^+^ T cell subsets according to titer (samples and boxplots as in (a)). (**d**) Pretreatment FOXP3^+^CD25^+^ T_regs_ cells are depleted in both CMV^+^_High titer_ and CMV^+^_Low titer_ groups relative to CMV^−^ patients (n=98, P_High titer versus CMV_−=0.0022, Est_adj_=−1.2 and P_Low titer versus CMV_−=0.0023, Est_adj_=−0.99) when adjusting for treatment regimen, tumor type, age, and sex (results from a linear mixed model random effects analysis, center point marks the estimate for the variable in association with FOXP3^+^CD25^+^ T_regs_ proportion of total CD4^+^ T cells, error bars mark the 95% CI, p values derived from ANOVA). ANOVA, analysis of variance; T_CM_, Central Memory T cells; T_EM_, Effector Memory T cells; T_EMRA_, Terminally Differentiated Effector Memory T cells Re-expressing CD45RA; T_Naive_, Naive T cells; T_regs_, Regulatory T cells. *P* < 0.05, **P < 0.01, ***P < 0.001, ****P < 0.0001.

To validate the hypothesis that CMV^+^_High titer_ patients have an increased proportion of their CD8^+^ T cell repertoire occupied by cytotoxic T cells, we performed operator-blinded flow cytometry, assessing major pretreatment subsets (n=143). Interestingly, we found that the well-described CMV-related changes in T cell subset composition^29^ were exacerbated in CMV^+^_High titer_ patients, with further skewing toward expansion of terminally differentiated effector memory cells (T_EMRA_) (p=0.0062) and a reciprocal reduction in the proportion of T_Naïve_ cells (p=0.016) compared with CMV^+^_Low titer_patients (figure 6a) in keeping with studies performed in healthy donors.^30^ An expansion of T_EMRA_ is associated with increased effector function and is a hallmark of memory inflation, thus we postulated that the proportion of CMV-reactive T cells may directly relate to anti-CMV IgG titer. Using a smaller cohort of CMV^+^ patients who were genotyped for HLA-A*02:01 carriage (n=25), we assessed the proportion of CD8^+^ T cells that were reactive to a multimer loaded with the inflationary CMV pp65 epitope. Interestingly, despite the association with increased total CD8^+^ T_EMRA_ count, we found no correlation between titer and the proportion of CMV-reactive CD8^+^ T cells binding this multimer (r=−0.0065, p=0.98). This indicates that the non-multimer-recognizing T_EMRA_ cells may cross-react to alternative inflationary CMV epitopes or/and additionally that the increased expansion of the T_EMRA_ compartment with increasing anti-CMV IgG titer reflects similar expansion of non-CMV specific clones, indicating a bystander effect of titer (figure 6b).

Prior studies have suggested that CMV has profound immunomodulatory effects on CD4^+^ T cells, with a population of CD4^+^ T_EMRA_ being associated with CMV independently of age.^31 32^ To explore the relationship between anti-CMV IgG titer and CD4^+^ T cells, we assessed major subsets using the same flow cytometry panel described for CD8^+^ T cells. We noted a significant expansion of the effector memory (T_EM_) compartment in the CMV^+^_High titer_ compared with CMV^+^_Low titer_ patients (p=0.009); however, a consistent direction of effect was noted for T_Naïve_ and T_EMRA_ subsets to that observed in CD8^+^ T cells, suggesting that the enhanced cytotoxicity associated with an elevated titer is true in helper T cells, corroborating data from healthy donors in the context of cancer.^33^ Finally, CMV^+^ patients were found to have reduced counts of T_regs_ (FOXP3^+^CD25^+^ regulatory T cells) in the periphery compared with CMV^-^ patients.^6^ We therefore assessed the relationship between T_reg_ proportion of total CD4^+^ T cells (n=98) in a multivariable analysis, correcting for tumor type, treatment intent, age, sex, and experimental batch. We found that increasing age at onset of systemic treatment was associated with a slight increase in T_reg_ proportion (p=0.037, Est_adj_=0.021), reinforcing the dichotomous effects of CMV from age-related immunosenescence.^34 35^ Interestingly, however, both CMV^+^_Low titer_ and CMV^+^_High titer_ patients had a reduction of their T_reg_ compartment compared with CMV^−^ patients (P_High titer vs CMV^−^_=0.0022, Est_adj_=−1.2 and P_Low titer vs CMV_^_−_^=0.0023, Est_adj_=−0.99). Notably, we observed no difference in T_reg_ proportion within CMV^+^ patients (p=0.57), indicating the effect of CMV on T_regs_ was influenced primarily by serostatus as opposed to total anti-CMV IgG titer.

### Clonal flux following ICB differs according to CMV titer

**Figure 7:**
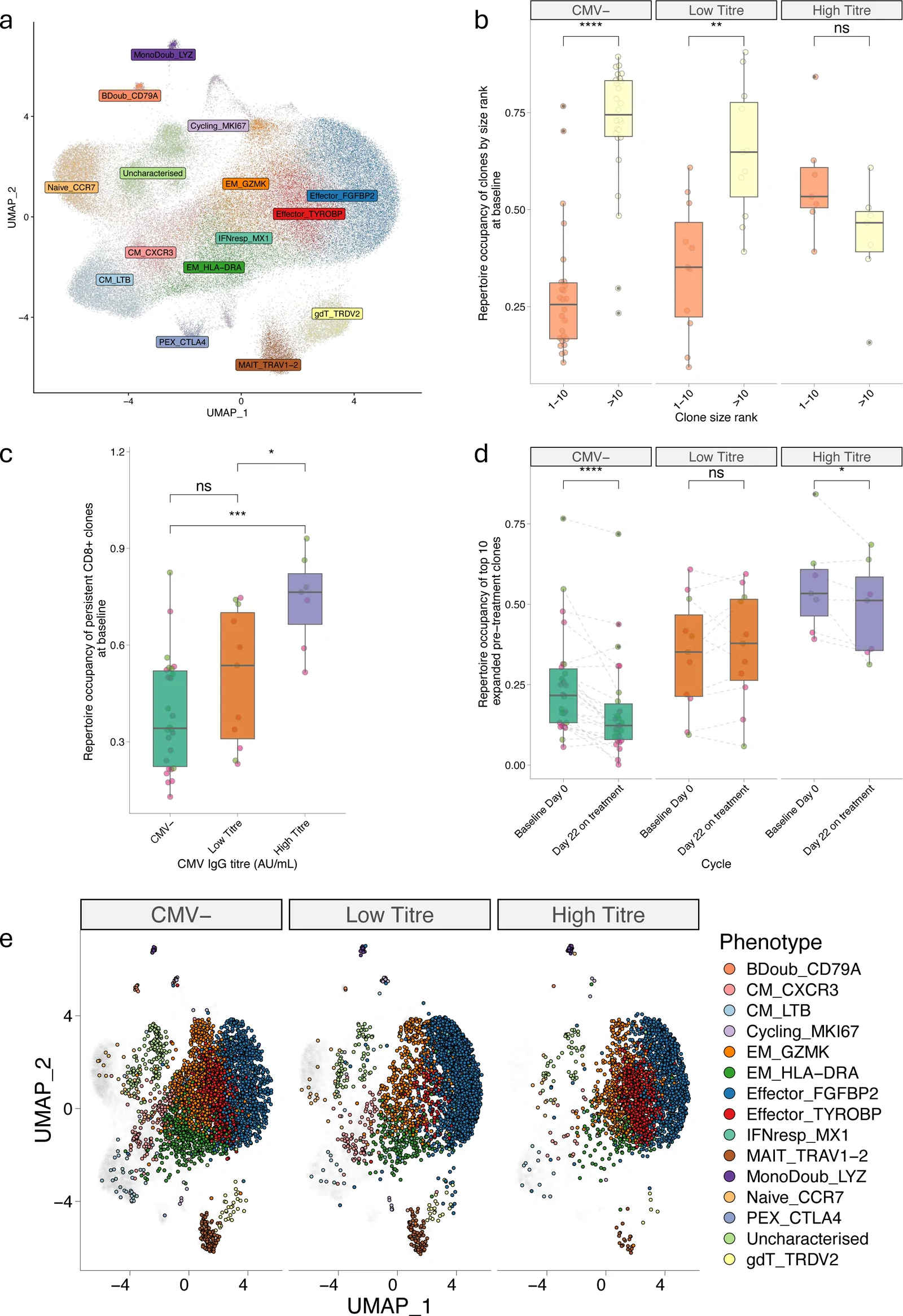
Altered clonal persistence according to titer following ICB treatment. (**a**) Uniform Manifold Approximation and Projection for Dimension Reduction (UMAP) plot of 111 890 CD8^+^ T cells from 44 paired pretreatment and post-treatment metastatic melanoma samples. Key marker genes for subset annotation are stated in the subset name. (**b**) Repertoire occupancy of the ten largest pretreatment clones per sample compared with all other clones identified according to CMV IgG titer (n_CMV_−=26, n_Low titer_=11, n_High titer_=7, p values derived from Kruskal-Wallis and post hoc Dunn tests). Lower and upper box hinges represent the 25th to 75th percentiles, the central line represents the median and whiskers extend to the highest and lowest values no greater than 1.5× IQR. (**c**) Baseline repertoire occupancy of persistent clones according to titer. Pink dots represent patients treated with cICB while green dots represent patients treated with sICB. P values and boxplots as in (b). (**d**) Change in repertoire occupancy of the ten largest persistent baseline clones according to titer. Colors represent the same treatment groups as in (c). While p values and boxplots are as in (b). (**e**) UMAP plot highlighting the phenotypes of cells belonging to the ten largest baseline clones in each patient. BDoub, B cell doublets; CM, central memory T cells; EM, effector memory T cells; gdT, gamma-delta T cells; IFNresp, interferon-responsive T cells; MAIT, mucosal-associated invariant T cells; MonoDoub, monocyte doublets; PEX, progenitor-exhausted T cells; sICB, single immune checkpoint blockade. *P* < 0.05, **P < 0.01, ***P < 0.001, ****P < 0.0001.

**Figure 8:**
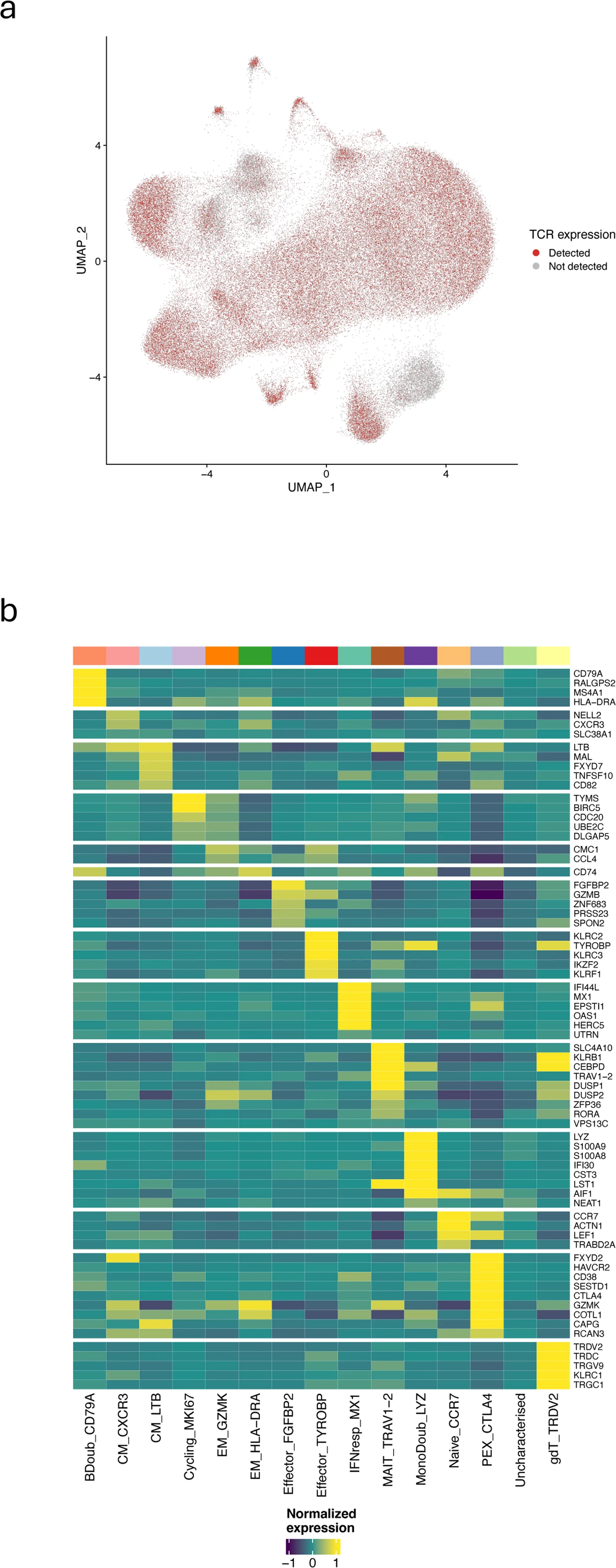
CD8^+^ T cell phenotypes in ICB-treated patients with metastatic melanoma. (**a**) UMAP of TCR detection across all cells (n=111 890 cells). (**b**) Heatmap showing the normalized gene expression of the top five marker genes for each subset by log2FC. Key marker genes for subset annotation are stated in the subset name. BDoub, B cell doublets; CM, central memory T cells; EM, effector memory T cells; gdT, gamma-delta T cells; ICB, immune checkpoint blockade; IFNresp, interferon-responsive T cells; MAIT, mucosal-associated invariant T cells; MonoDoub, monocyte doublets; PEX, progenitor-exhausted T cells; UMAP, Uniform Manifold Approximation and Projection.

To assess whether the profound skewing toward increased numbers of terminally differentiated CD8^+^ T cells observed in CMV^+^_High titer_ individuals was associated with expansion of specific T cell receptor (TCR) clonotypes, we investigated the effects of CMV titer in single-cell RNA/V(D)J sequencing data. Following unsupervised clustering, 15 transcriptomically distinct CD8^+^ T cell subsets were identified from 44 paired metastatic melanoma samples precheckpoint and postcheckpoint immunotherapy (n_CMV_-=26, n_Low titer_=11 and n_High titer_=7) (figure 7a). TCR mapping was used to identify the most expanded clones in each patient’s baseline sample, revealing that, in CMV^+^_High titer_ individuals, the ten largest clones occupied a comparable proportion of the peripheral repertoire to all other clones combined (p=0.073, figures 7b and 8a) with a trend toward increased occupancy of hyperexpanded clones. This was not observed in CMV^+^_Low titer_ or CMV^−^ patients (P_Low titer_=0.001, P_CMV^−^_=1×10^−9^). Secondarily, clones that persisted on immunotherapy had greater baseline repertoire occupancy in CMV^+^_High titer_ individuals relative to CMV^−^ (p=0.00012) and CMV^+^_Low titer_ individuals (p=0.02), suggesting that clonal turnover is diminished with increasing anti-CMV titer (figure 7c). Interestingly, when assessing persistence of the ten largest clonotypes pretreatment, we found that their repertoire occupancy diminished significantly in CMV^−^ (p=1.1×10^−5^) and CMV^+^_High titer_ patients (p=0.047) while remaining stable in CMV^+^_Low titer_ patients (p=0.46) (figure 7d). Lower clonal stability of the very largest clones among CMV^+^_High titer_ individuals relative to their CMV^+^_Low titer_ counterparts led us to investigate whether titer influenced the pretreatment phenotypes of these clones. Supporting this hypothesis, we found that hyperexpanded clones in CMV^+^_High titer_ patients were phenotypically more similar to those found in CMV^−^ patients, with an over-representation of cells in the *TYROBP*-expressing effector cluster with elevated expression of *CMC1*, a marker of exhaustion and mitochondrial dysfunction, and NK function (*KLRC2* & *KLRC1*) (figures 7e and 8b).^36^ Meanwhile, CMV^+^_Low titer_ patients had profound skewing of their largest clones toward *FGFBP2*-expressing effector cells, a subset that has been previously described to be beneficial following ICB treatment.^37^ This suggests that an amplified humoral response against CMV leads to an accumulation of dysfunctional effector cells with increased innate functional rewiring.

## Discussion

Here, in an extended study consisting of two cohorts of prospectively recruited patients receiving ICB for cancer, we replicate our previous observation from a less-mature dataset (n=308) of a protective effect of CMV serostatus against grade 3+ irAEs in a larger pan-cancer dataset that was divided into a discovery and internal validation cohort following age, sex, CMV, and treatment matching. In this new analysis, we further explore the relationship between the titer of CMV-reactive IgG antibodies and irAE risk across both cohorts (n=472). Consistent with prior studies, we find the anti-CMV IgG titer remains stable over time in CMV^+^ individuals,^10 11^ and anti-CMV IgG titer is independent of covariates including sex, age, and cancer type. Notably, while CMV^+^ patients have reduced risk of severe (grade 3+) irAEs, we observe that those with above median anti-CMV IgG titers are further protected against severe irAEs. Although this protection was most notable in recipients of anti-CTLA-4-containing cICB, which has a markedly increased risk of irAEs, a similar protection was seen in the smaller group of sICB recipients (p=0.041), where our power to detect protection was much reduced. This suggests that high anti-CMV IgG titer is not only associated with limited priming of autoreactive immunity following anti-CTLA-4 but also limits non-specific anti-PD-1 responses.^38^

Intriguingly, the protection attributable to high anti-CMV IgG titer is a composite of organ-specific effects, with the CMV-related protection for non-colitis irAEs being confined to those with above median anti-CMV IgG titers. Conversely, the protective effect of CMV seropositivity against the development of colitis was independent of anti-CMV IgG, indicating colitis is the most CMV-sensitive irAE. This observation provides further evidence for the risk factors of irAEs being organ-specific, inferring that pan-organ irAE studies have reduced sensitivity to detect both immunological and genetic associations. Moreover, our findings suggest that anti-CMV IgG titer reflects anatomical segregation of CMV effects on immunity, in accordance with studies describing organ-specific T cell responses to CMV infection. The cause of this is unclear, although in murine models, the titer of anti-CMV IgG relates to the magnitude of primary CMV infection.^7 39 40^

Further analysis of the relationship between anti-CMV IgG titer and circulating T cell subsets demonstrated that CMV^+^_High titer_ patients had more pronounced cytotoxic responses compared with CMV^+^_Low titer_ patients across both CD4^+^ and CD8^+^ T cell compartments, reflecting greater repertoire occupancy of T_EMRA_ cells. This finding corroborates prior work demonstrating synergism between T cell and humoral anti-CMV immunity.^33^ Interestingly, however, we did not observe a significant association between titer and the proportion of CMV-reactive CD8 against the major tegument protein pp65, suggesting that humoral immunity is either linked to bystander T cell activation or that this association is restricted to CMV-specific CD4^+^ T cells, as previously suggested.^33 41^ Antigen-independent activation has been reported following several viral infections, specifically by means of IL-15 signaling, and this is an avenue that requires further exploration in the context of cancer immunotherapy.^42 43^ Furthermore, we find that although there is no significant difference in the proportion of circulating T_regs_ according to titer, CMV and age appear to have dichotomous effects on this compartment, consistent with prior murine and human studies highlighting an accumulation of T_regs_ with enhanced immunosuppressive capacity with age.^44 45^ Despite T_regs_ being a core component of peripheral tolerance, studies in colitis patients have found increased T_reg_ infiltration in the colon compared with controls.^46^ Our work suggests that increasing circulating T_regs_ may be a marker of baseline inflammation in cancer and thus may aid in identifying patients who are at greater risk of developing toxicities, especially colitis.

Next, we investigate the clonal makeup of the CD8^+^ T cell compartment according to anti-CMV titer. We find that the largest clones occupy approximately half of the peripheral repertoire in CMV^+^_High titer_ patients and find greater clonal persistence of all clones following immunotherapy. Tracing hyperexpanded clonotypes through treatment, we find that they are less stable in CMV^+^_High titer_ and CMV^−^ individuals relative to their CMV^+^_Low titer_ counterparts. We find that this difference is associated with divergent phenotypes at baseline with increased markers of innate signaling and dysfunction in CMV^+^_High titer_ compared with CMV^+^_Low titer_ patients. While we do not propose that these large effector T cells directly mediate protective effects against irAEs in CMV^+^ individuals, we anticipate that such phenotypic skewing reflects a reciprocal depletion of smaller central memory T cells, corroborated by our flow cytometry data, with high activation and checkpoint expression that have previously been implicated in irAE development.^47^

Our study incorporates the largest analysis of the relationship between CMV status and irAE occurrence following ICB treatment. Nonetheless, although the cohort is prospectively sampled, our findings are observational in nature. While we do not find a change in CMV IgG titer following ICB treatment, this subanalysis included a small subset of OxCITE patients and increased sample size would be required to investigate this further. Whereas we previously noted no detectable CMV viraemias reflecting reactivation events in patients receiving ICB,^6^ we cannot exclude subthreshold detection as a surrogate for anti-CMV IgG titer. Furthermore, CMV reactivation may occur in immunosuppressed individuals and is a clinically relevant concern. Notwithstanding this, while we cannot exclude viral reactivation following steroid administration being a determinant of clinical outcome, we are not aware of clinically detected CMV reactivation in any of the cases in this cohort. We therefore think this is a relatively rare event, and any relationship between reactivation and CMV IgG titer remains unexplored. We are also limited currently to analysis of peripheral immunity and, while this is arguably the most informative tissue compartment from the perspective of biomarkers, further dissection of anti-CMV IgG titer on irAE development will require tissue-directed work.

Development of irAEs forms the leading cause of patient morbidity during ICB treatment and may shape clinical decision processes.^48^ Therefore, prospective, pretreatment biomarkers are of high value. This work indicates that not only can CMV serostatus, an easily determined and affordable parameter, be used to guide propensity for grade 3+ irAEs, but the anti-CMV IgG titer provides further clinical information with highly significant immune correlates. Specifically, patients who are seropositive for CMV are significantly less likely to develop colitis, irrespective of the anti-CMV IgG titer. Conversely, CMV protection against non-colitis irAEs is primarily conferred to those with an anti-CMV IgG titer of ≥186 a.u.mL^−1^. The titer of anti-CMV IgG may have translational relevance regarding the management of patients developing irAEs, and CMV serology is a rapid baseline predictor of irAE risk that has easy scalability to a standard-of-care testing in a clinical setting. Our data suggest that in CMV^−^ patients developing colitis, early escalation to steroid-sparing biologics may be necessitated irrespective of the titer. Conversely, for non-colitis irAEs, this approach may be valid in both CMV^−^ and CMV^+^_Low titer_ patients. However, we believe ICB choice should primarily be guided by purported clinical benefit, and here our previous observation regarding increased risk of death in CMV^−^ patients receiving anti-PD-1 alone, but not in combination with anti-CTLA-4, is most pertinent.^6^ It is important to acknowledge that formal changes in treatment guidelines will require more studies, but these data further support a crucial relationship between CMV status and response to ICB in melanoma.

## Methods

### Patients and clinical outcomes

Patients were recruited between November 23, 2015 and February 6, 2026. Patients eligible for ICB in the Oxford University Hospitals NHS Foundation Trust were recruited prospectively. These included patients with advanced or metastatic melanoma (n=325), renal cell carcinoma (n=24), microsatellite instable colorectal cancer (n=18), mesothelioma (n=26), and cutaneous squamous cell carcinoma (n=14). Patients due to receive adjuvant ICB for melanoma (n=57) and renal cell carcinoma (n=8) were also eligible. Treatment regimens are listed in online supplemental material. irAEs were assessed according to the National Cancer Institute’s Common Terminology Criteria for Adverse Events, version 5.0. Hematological data were extracted from electronic records and primarily confined to those patients within the Oxford University Hospitals Trust catchment area. Lymphocyte count and neutrophil:lymphocyte ratio were determined using a Sysmex XN series analyzer in an NHS clinical diagnostic laboratory. These values were determined from blood counts taken 20–50 days after the patient’s first cycle of immunotherapy (median 35 days after treatment, IQR: 22–50 days). Discovery (n=250) and validation (n=222) cohorts were defined according to a bioinformatic matching process accounting for age decile, sex, CMV serostatus, and treatment. Regarding the original cohort defined in our previous study, 168/308 patients were included in the discovery cohort and 140/308 were included in the validation cohort. Median follow-up for discovery and validation cohorts was 40.8 and 39.6 months, respectively.

### Sample collection

Up to 50 mL of whole blood was collected from each patient in EDTA vacutainer tubes, immediately prior to the first and second doses of ICB. Plasma and peripheral blood mononuclear cells (PBMCs) were separated after blood collection via density centrifugation using Ficoll-Paque (Cytiva). Anti-CMV IgG antibody titers were determined from pretreatment plasma that was diluted 1:2 in HBSS on an Abbott Architect i2000 at the John Radcliffe Hospital, Oxford University Hospitals. Patients with an antibody titer of <4 a.u.mL^−1^ were called CMV^−^. For CMV+ patients saturating values (*>*250 a.u.mL^−1^) were called as 250 a.u.mL^−1^, median value=92.9 a.u.mL^−1^ (mean=109.3 a.u.mL^−1^, IQR: 54.4–161.2 a.u.mL^−1^). CD8^+^ T cells were isolated from PBMCs for bulk RNA sequencing using Miltenyi Biotech magnetic separation via positive selection as per the manufacturer’s instructions. PBMCs were counted and stored in liquid nitrogen at 5–10×10^6^ cells/vial.

### Nucleic acid extraction

Positively selected CD8^+^ T cells (bulk RNA sequencing) and PBMCs (genotyping) were spun down at 4°C and resuspended in 350 µL of RLTplus buffer (Qiagen) supplemented with 2% DTT and stored at −80°C for DNA/RNA extraction. Samples were homogenized using the QIAshredder kit (Qiagen). DNA/RNA was extracted using the AllPrep Universal DNA/RNA/miRNA Kit (Qiagen). DNase I was used during the RNA extraction protocol to minimize DNA contamination. RNA was eluted into RNase-free water and quantified via Qubit analysis. Samples were stored at −80°C until library preparation.

### Bulk RNA-sequencing

RNA was thawed on ice, and messenger RNA was isolated using the NEBNext Poly(A) mRNA Magnetic Isolation Module Kit. Library preparation was performed using NEBNext Ultra II Directional RNA Library Prep Kit for Illumina. mRNA was then sequenced using 150-base pair (bp)-end sequencing on an Illumina NovaSeq 6000/NovaSeq XPlus or 75bp-end sequencing on an Illumina HiSeq-4000, at Genewiz and the Oxford Genome Centre, respectively. Reads were aligned to the hg38 build of the human genome using HISAT2, and read count data was generated using HTSeq.^49 50^ High-quality reads were chosen based on the MAPQ score provided by bamtools.^51^ Marking and removal of duplicated reads was performed using Picard, while samtools was used to calculate statistics.^52^ DESeq2 (v.1.48.1) was used to perform differential expression analysis between CMV^+^_Low titer_, CMV^+^_High titer_ and CMV^−^ patients’ pretreatment samples.^53^ Age, sex, tumor type, treatment intent, and the first principal component of all genes were adjusted for in the design formula and only genes with a mean expression >10 were included in the analysis. Normalized expression of *IFNG*, *KLRD1*, *TBX21* and *ZNF683* was also determined using DESeq2.^53^

NovaSeq 6000/NovaSeq XPlus or 75bp-end sequencing on an Illumina HiSeq-4000, at Genewiz and the Oxford Genome Centre, respectively. Reads were aligned to the hg38 build of the human genome using HISAT2, and read count data was generated using HTSeq.^49 50^ High-quality reads were chosen based on the MAPQ score provided by bamtools.^51^ Marking and removal of duplicated reads was performed using Picard, while Samtools was used to calculate statistics.^52^ DESeq2 (v.1.48.1) was used to perform differential expression analysis between CMV^+^_Low titer_, CMV^+^_High titer_ and CMV^-^ patients’ pretreatment samples.^53^ Age, sex, tumor type, treatment intent, and the first principal component of all genes were adjusted for in the design formula, and only genes with a mean expression >10 were included in the analysis. Normalized expression of *IFNG*, *KLRD1*, *TBX21,* and *ZNF683* was also determined using DESeq2.^53^

### Flow cytometry

Cryopreserved patient PBMC samples were thawed at 37°C and washed with HBSS. 1×10^6^ PBMCs were plated, and viability staining was performed with eBioscience Fixable Viability Dye eFluor780 (Invitrogen, cat. no. 65-0865-14) or Fixability Viability Stain 440UV (BD Biosciences) for 30 min at 4°C. PBMCs were washed with HBSS supplemented with 5% FCS and BD Horizon Brilliant Stain Buffer (BD Biosciences, 563794). Staining for T cell surface markers was performed for 30 min at 4°C using antibodies directed against markers listed in online supplemental material. PBMCs were washed once in cell staining buffer (BioLegend, cat. no. 420201) and incubated with Streptavidin (BV785, BioLegend cat. no. 405249) for 30 min at 4°C for IgG_4_ staining. Following surface staining, PBMCs were permeabilized with Foxp3/Transcription Factor Staining Buffer Set (Invitrogen, cat. no. 00-5523-00) for intranuclear staining or Transcription Factor Buffer Set (BD Biosciences, cat. no. 562574) for cytoplasmic staining, for 30 min, and incubated with intracellular staining antibodies listed in online supplemental material. PBMCs were resuspended in 2% paraformaldehyde, and acquisition was performed on a FACSSymphony A5 Cell Analyzer (BD Biosciences) or a Fortessa X-20. The CMV-tetramer panel was acquired on an Attune NxT. Data were analyzed using FlowJo V.10.7.1 (BD Biosciences).

### HLA-typing

Genotyping was performed using the Illumina Global Screening Array 24 V.3. The vcf files for chromosome 6 were input into the Michigan Imputation Server Genotype Imputation HLA (Minimac4) 1.5.8 pipeline against the Multi-ethnic HLA reference panel. Results were filtered by class I and II genes.

### Single-cell RNA/V(D)J sequencing

A combination of PBMC (n=39 samples), CD3^+^-sorted (n=14 samples), and positively selected CD8^+^ T cells (n=35 samples) were counted; equal counts were pooled for 5–8 samples from distinct patients. Pools were oil-partitioned into single-cell droplets using a 10X Chromium System. A total of 60 000 cells were loaded onto each partitioning reaction, followed by cell lysis and reverse transcription. 5’ GEX and V(D)J libraries were made from complementary DNA using the Chromium Next GEM Single Cell 5’ Reagent Kit v2 (Dual Index). Sequencing was performed on a NovaSeq6000 (Illumina): 150(bp) paired-end reads for both 5’ RNA libraries and V(D)J libraries to a depth of 25 000 reads per cell. FASTQ files were generated from raw Illumina BCL outputs using Cellranger (V.6.0.1) for GEX libraries and Cellranger VDJ for V(D)J libraries. For quality control, the R package scater^54^ was used to identify single-cell outliers, and the R package scran was used to identify biological doublets. Cells were allocated to individual patients using vireoSNP^55^ and cellsnp-lite,^56^ while genotypic doublets were removed using scrublet.^57^ CD8^+^ T cells were then filtered for expression of *CD3D* and either *CD8A* or *CD8B,* along with absence of *CD14* expression. Seurat^58^ was used to remove cells with <500 total unique molecular identifiers, *<*300 features, or a mitochondrial gene percentage >20%. Genes expressed in <5 cells were also excluded. Remaining cells were subjected to Harmony^59^ integration to correct for batch effects prior to dimensionality reduction and uniform manifold approximation and projection clustering. Cell clusters were generated between resolutions of 0.8 and 2.0 and assessed using the R package clustree,^60^ with values of 1.0 demonstrating high cluster stability. Cells were assigned to subsets based on curated transcripts per million normalized counts for CD8^+^ T cells from previous public cancer scRNA-seq datasets using SingleR^61^ and refined using manual annotation.

### Statistical analysis

Statistical analysis was performed using R V.4.5.1. Cumulative incidence and competing risk regression were performed using tidycmprsk (v1.1.0) to account for guarantee-time bias. Univariate estimates were calculated for time to irAE development using a semicompeting risks model, where death was the competing risk. Significance testing was performed using Gray’s test. Competing risk regression was used to calculate the Fine-Gray subdistribution hazard for the association of CMV titer and each irAE. The optimum titer cut-off for predicting the risk of severe non-colitis irAEs was determined using two methods, yielding the same threshold. First, a time-independent ROC analysis was performed, where patients who died prior to developing a toxicity were censored. The second used a sliding window of titers and assessed the predictive value of each cut-off using a univariate competing risk regression, as described above. Median follow-up time was calculated using the reverse Kaplan-Meier method described by Schemper and Smith.^62^ Distributions were compared using either a Wilcoxon signed-rank test for paired data or a Wilcoxon rank-sum test for unpaired data. For multiple comparisons, a Kruskal-Wallis test was used, followed by a post hoc Dunn test. Correlation analysis was performed using a Spearman’s rank correlation test. Estimates for FOXP3^+^CD25^+^T_reg_ proportions were obtained using a linear mixed effects model using the lme4 package (V.1.1-37), where batch was modeled as a random effect. All multivariable analyses included age, sex, treatment regimen, and treatment intent as covariates unless stated otherwise. Plots were generated using ggplot2 (V.3.5.2). For all data, *p*<*0.05, **p*<*0.01, ***p*<*0.001, ****p*<*0.0001.

## Supplementary material

10.1136/jitc-2026-016050 online supplemental file 1

10.1136/jitc-2026-016050 online supplemental file 2

10.1136/jitc-2026-016050 online supplemental file 3

## Data Availability

Data are available on reasonable request.
