# Supplementary material for: Anti-CMV IgG titer determines organ-specific protection toward immune checkpoint blockade-induced toxicities
Source: J Immunother Cancer. 2026 Sep 7;14(9):e016050. doi: 10.1136/jitc-2026-016050 (PMC13560976; doi:10.1136/jitc-2026-016050)
Supplement: Supplementary file 1 [file jitc-14-9-s001.pdf]

Cancer	Treatment Intent	Combination treatment	Treatment regimen
Melanoma	Adjuvant	No	pembrolizumab 200mg q3w or 400mg q6w/ nivolumab 240mg q2w or 480mg q4w
Melanoma	Palliative	No	pembrolizumab 200mg q3w or 400mg q6w/ nivolumab 240mg q2w or 480mg q4w
Melanoma	Palliative	Yes	ipilimumab 3mg/kg q3w plus nivolumab 1mg/kg q3w for up to 4 doses followed by maintenance nivolumab
RCC	Adjuvant	No	pembrolizumab 600mg q6w for up to 1 year
RCC	Palliative	Yes	ipilimumab 1mg/kg plus nivolumab 3mg/kg q3w for up to 4 doses followed by maintenance nivolumab 480mg q4w for up to 2 years
Mesothelioma	Palliative	Yes	ipilimumab 1mg/kg q6w plus nivolumab 360mg q3w for up to 2 years
cSCC	Palliative	No	cemiplimab 350mg q3w for up to 2 years
Colorectal	Palliative	No	pembrolizumab 200mg q3w or 400mg q6w
Colorectal	Palliative	Yes	ipilimumab 1mg/kg plus nivolumab 3mg/kg q3w for up to 4 doses followed by maintenance nivolumab 480mg q4w for up to 2 years

Supplementary table 1- Treatment regimens in OxCITE study

Phenotyping panel 1- Fortessa X-20

Laser	Detector	Target	Fluorochrome	Dilution	Clone	Source	Cat number	Verified reactivity	Validation
355	379/28	CD27	BUV395	50	O323	BD Biosciences	751677	Human	Flow Cytometry
355	515/30	CD3	BUV496	50	UCHT1	BD Biosciences	612940	Human	Flow Cytometry
355	740/35	CD8	BUV737	50	SK1	BD Biosciences	612754	Human, Rhesus, Cynomolgus, Baboon	Flow Cytometry
405	450/50	CD56	BV421	50	5.1H11	Biolegend	362552	Human	FC quality tested
405	525/50	CD4	BV510	50	RPA-T4	Biolegend	300546	Human	FC quality tested
405	610/20	CD25	BV605	50	M-A51	Biolegend	356142	Human	FC quality tested
405	670/30	PD-1	BV650	50	EH12.2H7	Biolegend	329950	Human	FC quality tested
405	780/60	CD28	BV786	10	CD28.2	Biolegend	302950	Human, Cynomolgus, Rhesus	FC quality tested
488	530/30	CD19	FITC	30	HIB19	Biolegend	302206	Human	FC quality tested
488	695/40	CD45RA	PerCP-Cy5.5	20	HI100	Biolegend	304122	Human	FC quality tested
561	585/15	IgG4-Strep	PE	250/200		Invitrogen	MH1542	Human	
561	610/20	KI-67	PE-Dazzle594	80	Ki-67	Biolegend	350533	Human	ICFC quality tested
561	780/60	CCR7	PE-Cy7	20	G043H7	Biolegend	353226	Human	FC quality tested
640	670/30	FoxP3	APC	75	236A/E7	Invitrogen	17-4777-42	Human, Non-human primate, Rhesus	Flow Cytometry
640	730/45	CD127	AF700	50	A019D5	Biolegend	351343	Human	FC quality tested
640	780/60	Viability	APC-Cy7	1000		Invitrogen	65-0865-14		

CMV multimer panel- Attune NxT

Laser	Detector	Target	Fluorochrome	Dilution	Clone	Source	Cat number	Verified reactivity	Validation
640	780/60	Viability	APC-Cy7	1000		Invitrogen	65-0865-14		
405	710/50	CD3	BV711	50	UCHT1	Biolegend	300463	Human	FC quality tested
488	695/40	CD8	PerCP-Cy5.5	20	HIT8a	Biolegend	300923	Human	FC quality tested
640	670/14	CCR7	APC	20	G043H7	Biolegend	353213	Human	FC quality tested
405	450/50	CD45RA	BV421	50	HI100	Biolegend	304130	Human	FC quality tested
561	585/16	CMV multimer	PE	160		Miltenyi	130-134-623		

Phenotyping panel 2- FACSSymphony A5 Cell Analyser (BD Bioscience)

Laser	Detector	Target	Fluorochrome	Dilution	Clone	Source	Cat number	Verified reactivity	Validation
488	530/30	TIGIT	KirBlue	50	A15153G	BioLegend	372731	Human	FC quality tested
488	710/50	CXCR3	PerCP/Cy5.5	150	G025H7	BioLegend	353713	Human, Cynomolgus, Rhesus	FC quality tested
640	670/30	FOXP3	APC	75	FJK-16s	Invitrogen	17-5773-82	Bovine, Dog, Cat, Mouse, Pig, Rat	Flow cytometry
640	730/45	CCR6	AF700	100	G034E3	BioLegend	353433	Human, Cynomolgus, Rhesus	FC quality tested
640	780/60	CXCR5	APCCY7	75	J252D4	BioLegend	356925	Human	FC quality tested
405	450/50	CD103	BV421	125	2.00E+07	BioLegend	121421	Mouse	FC quality tested
405	525/50	CD39	BV510	50	A1	BioLegend	328219	Human, Cynomolgus, Rhesus	FC quality tested
405	610/20	CD127	BV605	125	HIL-7R-M21	BD Biosciences	562662	Human	Flow cytometry
405	670/30	PD1	BV650	50	EH12.1	BD Biosciences	564104	Human	Flow cytometry
405	710/50	Ki67	BV711	100	B56	BD Biosciences	563755	Human, Mouse, Rat, Rhesus	Flow cytometry
405	810/40	IgG4-biot	BV785	250		Streptavidin from BioLegend	405249	All species	ICFC verified and FC quality tested
561	585/15	CCR8	PE	50	L263G8	BioLegend	360603	Human	FC quality tested
561	610/20	CCR4	PEDazzle594	125	L291H4	BioLegend	359419	Human	FC quality tested
561	780/60	CTLA4	PECy7	75	L3D10	BioLegend	349913	Human	ICFC quality tested and Fc verified
355	379/28	CD27	BUV395	50	L128	BD Biosciences	563816	Human	Flow cytometry
355	450/50	viability	FVS440UV						
355	515/30	CD3	BUV496	150	UCHT1	BD Biosciences	612941	Human	Flow cytometry
355	580/20	CD45RA	BUV563	150	HI100	BioLegend	304133	Human	FC quality tested
355	605/20	CD25	BUV615	50	2A3	BD Biosciences	612997	Human	Flow cytometry
355	735/30	CD8	BUV737	500	SK1	BD Biosciences	612755	Human, Cynomolgus, Rhesus, Baboon	Flow cytometry
355	810/40	CD4	BUV805	200	SK3	BD Biosciences	612888	Human	Flow cytometry

Supplementary Table 4- Flow cytometry panels

Age	Sex	cancer	Intent	Rx	Titre	Cohort	irAE data	Survival data	Lymph Count	NLR	Transcriptomics	Baseline Flow	Day 22 Flow	Treg Prop	CMV-multimer flow	Titre stability	Single-cell
53	Female	Melanoma	Palliative	sICB	CMV-	Discovery	TRUE	TRUE	TRUE	TRUE	FALSE	FALSE	TRUE	TRUE	FALSE	FALSE	FALSE
42	Male	Melanoma	Palliative	sICB	High Titre	Discovery	TRUE	TRUE	TRUE	TRUE	TRUE	FALSE	FALSE	FALSE	FALSE	FALSE	FALSE
47	Male	Melanoma	Palliative	sICB	High Titre	Validation	TRUE	TRUE	TRUE	TRUE	TRUE	FALSE	FALSE	FALSE	FALSE	FALSE	FALSE
87	Male	Melanoma	Palliative	sICB	Low Titre	Validation	TRUE	TRUE	TRUE	FALSE	TRUE	TRUE	FALSE	TRUE	FALSE	FALSE	FALSE
64	Female	Melanoma	Palliative	cICB	CMV-	Validation	TRUE	TRUE	TRUE	TRUE	TRUE	FALSE	FALSE	FALSE	FALSE	FALSE	FALSE
40	Female	Melanoma	Palliative	cICB	CMV-	Validation	TRUE	TRUE	TRUE	TRUE	TRUE	FALSE	FALSE	FALSE	FALSE	FALSE	FALSE
21	Female	Melanoma	Palliative	cICB	CMV-	Discovery	TRUE	TRUE	FALSE	FALSE	TRUE	FALSE	FALSE	FALSE	FALSE	FALSE	FALSE
79	Male	Melanoma	Palliative	sICB	CMV-	Discovery	TRUE	TRUE	TRUE	FALSE	TRUE	FALSE	TRUE	FALSE	FALSE	FALSE	TRUE
77	Female	Melanoma	Palliative	sICB	High Titre	Validation	TRUE	TRUE	TRUE	TRUE	TRUE	TRUE	TRUE	TRUE	FALSE	FALSE	FALSE
65	Male	Melanoma	Palliative	cICB	High Titre	Validation	TRUE	TRUE	TRUE	TRUE	TRUE	FALSE	FALSE	FALSE	FALSE	FALSE	FALSE
75	Female	Melanoma	Palliative	sICB	High Titre	Validation	TRUE	TRUE	TRUE	TRUE	TRUE	TRUE	TRUE	TRUE	FALSE	TRUE	FALSE
75	Male	Melanoma	Palliative	sICB	CMV-	Discovery	TRUE	TRUE	TRUE	TRUE	TRUE	FALSE	FALSE	FALSE	FALSE	FALSE	FALSE
59	Female	Melanoma	Palliative	cICB	Low Titre	Validation	TRUE	TRUE	TRUE	TRUE	TRUE	TRUE	TRUE	TRUE	FALSE	FALSE	TRUE
72	Female	Melanoma	Palliative	sICB	CMV-	Discovery	TRUE	TRUE	FALSE	FALSE	TRUE	TRUE	FALSE	TRUE	FALSE	FALSE	TRUE
71	Female	Melanoma	Palliative	sICB	CMV-	Validation	TRUE	TRUE	TRUE	TRUE	FALSE	TRUE	FALSE	TRUE	FALSE	FALSE	FALSE
70	Male	Melanoma	Palliative	cICB	CMV-	Validation	TRUE	TRUE	TRUE	FALSE	FALSE	TRUE	FALSE	TRUE	FALSE	FALSE	FALSE
68	Female	Melanoma	Palliative	sICB	CMV-	Discovery	TRUE	TRUE	TRUE	TRUE	TRUE	FALSE	FALSE	TRUE	FALSE	FALSE	FALSE
68	Female	Melanoma	Palliative	sICB	CMV-	Validation	TRUE	TRUE	FALSE	FALSE	TRUE	TRUE	FALSE	TRUE	FALSE	FALSE	TRUE
74	Female	Melanoma	Palliative	sICB	CMV-	Discovery	TRUE	TRUE	FALSE	FALSE	TRUE	TRUE	FALSE	TRUE	FALSE	FALSE	FALSE
70	Male	Melanoma	Palliative	sICB	Low Titre	Discovery	TRUE	TRUE	TRUE	FALSE	TRUE	TRUE	TRUE	TRUE	FALSE	FALSE	FALSE
44	Female	Melanoma	Palliative	cICB	CMV-	Validation	TRUE	TRUE	TRUE	TRUE	TRUE	FALSE	FALSE	FALSE	FALSE	FALSE	FALSE
68	Female	Melanoma	Palliative	sICB	High Titre	Discovery	TRUE	TRUE	TRUE	TRUE	TRUE	TRUE	TRUE	TRUE	FALSE	FALSE	FALSE
49	Male	Melanoma	Palliative	cICB	CMV-	Discovery	TRUE	TRUE	TRUE	TRUE	TRUE	FALSE	FALSE	FALSE	FALSE	FALSE	FALSE
63	Male	Melanoma	Palliative	cICB	Low Titre	Validation	TRUE	TRUE	TRUE	TRUE	FALSE	FALSE	TRUE	FALSE	FALSE	FALSE	FALSE
86	Male	Melanoma	Palliative	sICB	Low Titre	Discovery	TRUE	TRUE	TRUE	TRUE	TRUE	TRUE	FALSE	TRUE	FALSE	FALSE	FALSE
67	Male	Melanoma	Palliative	cICB	CMV-	Discovery	TRUE	TRUE	TRUE	TRUE	TRUE	FALSE	TRUE	FALSE	FALSE	FALSE	FALSE
47	Male	Melanoma	Palliative	cICB	CMV-	Discovery	TRUE	TRUE	TRUE	TRUE	TRUE	TRUE	FALSE	TRUE	FALSE	FALSE	FALSE

46	Male	Melanoma	Palliative	cICB	CMV-	Discovery	TRUE	TRUE	FALSE	FALSE	TRUE	FALSE	FALSE	FALSE	FALSE	FALSE	FALSE
67	Male	Melanoma	Palliative	cICB	High Titre	Discovery	TRUE	TRUE	TRUE	TRUE	TRUE	FALSE	FALSE	FALSE	FALSE	FALSE	FALSE
66	Male	Melanoma	Palliative	cICB	CMV-	Discovery	TRUE	TRUE	TRUE	FALSE	TRUE	TRUE	FALSE	TRUE	FALSE	FALSE	FALSE
66	Female	Melanoma	Palliative	cICB	CMV-	Discovery	TRUE	TRUE	TRUE	TRUE	TRUE	FALSE	FALSE	FALSE	FALSE	FALSE	FALSE
72	Male	Melanoma	Palliative	cICB	High Titre	Discovery	TRUE	TRUE	TRUE	TRUE	TRUE	FALSE	TRUE	FALSE	FALSE	TRUE	FALSE
67	Female	Melanoma	Palliative	sICB	CMV-	Validation	TRUE	TRUE	TRUE	TRUE	TRUE	TRUE	FALSE	FALSE	FALSE	FALSE	FALSE
80	Male	Melanoma	Palliative	sICB	Low Titre	Discovery	TRUE	TRUE	TRUE	TRUE	TRUE	TRUE	FALSE	TRUE	FALSE	FALSE	FALSE
72	Male	Melanoma	Palliative	sICB	Low Titre	Validation	TRUE	TRUE	TRUE	TRUE	TRUE	TRUE	FALSE	TRUE	FALSE	TRUE	TRUE
70	Male	Melanoma	Palliative	sICB	CMV-	Discovery	TRUE	TRUE	TRUE	FALSE	TRUE	TRUE	FALSE	TRUE	FALSE	FALSE	FALSE
83	Female	Melanoma	Palliative	sICB	High Titre	Discovery	TRUE	TRUE	TRUE	TRUE	TRUE	TRUE	FALSE	TRUE	FALSE	TRUE	FALSE
65	Female	Melanoma	Palliative	cICB	CMV-	Validation	TRUE	TRUE	TRUE	TRUE	FALSE	FALSE	FALSE	FALSE	FALSE	FALSE	FALSE
83	Female	Melanoma	Palliative	sICB	CMV-	Discovery	TRUE	TRUE	TRUE	TRUE	TRUE	TRUE	FALSE	TRUE	FALSE	FALSE	FALSE
84	Male	Melanoma	Palliative	sICB	High Titre	Discovery	TRUE	TRUE	TRUE	TRUE	TRUE	TRUE	TRUE	TRUE	FALSE	TRUE	TRUE
84	Female	Melanoma	Palliative	sICB	High Titre	Validation	TRUE	TRUE	FALSE	FALSE	TRUE	TRUE	FALSE	TRUE	FALSE	TRUE	TRUE
50	Male	Melanoma	Palliative	cICB	CMV-	Discovery	TRUE	TRUE	TRUE	TRUE	TRUE	FALSE	TRUE	FALSE	FALSE	FALSE	TRUE
71	Male	Melanoma	Palliative	sICB	CMV-	Validation	TRUE	TRUE	TRUE	TRUE	TRUE	TRUE	FALSE	TRUE	FALSE	FALSE	TRUE
67	Female	Melanoma	Palliative	sICB	CMV-	Discovery	TRUE	TRUE	TRUE	TRUE	TRUE	FALSE	TRUE	FALSE	FALSE	FALSE	FALSE
70	Male	Melanoma	Palliative	cICB	High Titre	Validation	TRUE	TRUE	TRUE	TRUE	TRUE	FALSE	TRUE	FALSE	FALSE	FALSE	TRUE
57	Male	Melanoma	Palliative	cICB	CMV-	Validation	TRUE	TRUE	TRUE	TRUE	TRUE	FALSE	TRUE	FALSE	FALSE	FALSE	TRUE
32	Female	Melanoma	Palliative	cICB	Low Titre	Validation	TRUE	TRUE	TRUE	TRUE	TRUE	FALSE	FALSE	FALSE	FALSE	FALSE	FALSE
81	Female	Melanoma	Palliative	sICB	High Titre	Validation	TRUE	TRUE	TRUE	TRUE	TRUE	FALSE	TRUE	FALSE	FALSE	FALSE	FALSE
82	Male	Melanoma	Palliative	sICB	High Titre	Discovery	TRUE	TRUE	TRUE	TRUE	TRUE	FALSE	FALSE	FALSE	FALSE	FALSE	TRUE
72	Female	Melanoma	Palliative	sICB	CMV-	Validation	TRUE	TRUE	TRUE	TRUE	FALSE	FALSE	FALSE	FALSE	FALSE	FALSE	TRUE
57	Male	Melanoma	Palliative	cICB	Low Titre	Validation	TRUE	TRUE	TRUE	TRUE	TRUE	FALSE	FALSE	FALSE	FALSE	TRUE	TRUE
45	Female	Melanoma	Palliative	cICB	Low Titre	Discovery	TRUE	TRUE	TRUE	FALSE	TRUE	FALSE	FALSE	FALSE	FALSE	TRUE	TRUE
43	Female	Melanoma	Palliative	sICB	Low Titre	Discovery	TRUE	TRUE	TRUE	TRUE	TRUE	FALSE	FALSE	FALSE	FALSE	FALSE	TRUE
56	Male	Melanoma	Palliative	cICB	CMV-	Discovery	TRUE	TRUE	TRUE	TRUE	TRUE	FALSE	FALSE	FALSE	FALSE	FALSE	TRUE
65	Female	Melanoma	Palliative	cICB	CMV-	Discovery	TRUE	TRUE	TRUE	TRUE	TRUE	FALSE	TRUE	FALSE	FALSE	FALSE	FALSE
68	Male	Melanoma	Palliative	cICB	CMV-	Validation	TRUE	TRUE	TRUE	TRUE	TRUE	TRUE	FALSE	TRUE	FALSE	FALSE	TRUE
69	Male	Melanoma	Palliative	sICB	CMV-	Discovery	TRUE	TRUE	FALSE	FALSE	TRUE	FALSE	FALSE	FALSE	FALSE	FALSE	TRUE

82	Male	Melanoma	Palliative	sICB	CMV-	Discovery	TRUE	TRUE	FALSE	FALSE	TRUE	TRUE	FALSE	TRUE	FALSE	FALSE	FALSE
66	Male	Melanoma	Palliative	cICB	CMV-	Validation	TRUE	TRUE	TRUE	TRUE	TRUE	FALSE	FALSE	FALSE	FALSE	FALSE	FALSE
47	Female	Melanoma	Palliative	cICB	CMV-	Discovery	TRUE	TRUE	TRUE	TRUE	TRUE	FALSE	FALSE	FALSE	FALSE	FALSE	FALSE
59	Female	Melanoma	Palliative	cICB	Low Titre	Discovery	TRUE	TRUE	TRUE	TRUE	TRUE	TRUE	FALSE	FALSE	FALSE	FALSE	FALSE
41	Female	Melanoma	Palliative	sICB	Low Titre	Validation	TRUE	TRUE	TRUE	TRUE	FALSE	FALSE	FALSE	FALSE	FALSE	FALSE	FALSE
81	Female	Melanoma	Palliative	sICB	High Titre	Discovery	TRUE	TRUE	TRUE	TRUE	TRUE	FALSE	TRUE	FALSE	FALSE	TRUE	TRUE
52	Male	Melanoma	Palliative	cICB	CMV-	Validation	TRUE	TRUE	FALSE	FALSE	TRUE	TRUE	FALSE	FALSE	FALSE	FALSE	FALSE
78	Male	Melanoma	Palliative	sICB	Low Titre	Validation	TRUE	TRUE	TRUE	TRUE	TRUE	FALSE	FALSE	FALSE	FALSE	FALSE	FALSE
70	Female	Melanoma	Palliative	cICB	CMV-	Discovery	TRUE	TRUE	TRUE	TRUE	TRUE	FALSE	TRUE	FALSE	FALSE	FALSE	FALSE
58	Female	Melanoma	Palliative	cICB	CMV-	Validation	TRUE	TRUE	TRUE	FALSE	TRUE	TRUE	FALSE	TRUE	FALSE	FALSE	FALSE
62	Female	Melanoma	Palliative	cICB	High Titre	Discovery	TRUE	TRUE	TRUE	TRUE	TRUE	FALSE	FALSE	FALSE	FALSE	FALSE	FALSE
58	Female	Melanoma	Palliative	cICB	Low Titre	Discovery	TRUE	TRUE	TRUE	TRUE	TRUE	FALSE	FALSE	FALSE	FALSE	FALSE	FALSE
71	Female	Melanoma	Palliative	sICB	CMV-	Discovery	TRUE	TRUE	TRUE	TRUE	TRUE	FALSE	FALSE	FALSE	FALSE	FALSE	FALSE
80	Female	Melanoma	Palliative	sICB	Low Titre	Validation	TRUE	TRUE	TRUE	FALSE	TRUE	FALSE	FALSE	FALSE	FALSE	FALSE	FALSE
59	Female	Melanoma	Palliative	cICB	CMV-	Validation	TRUE	TRUE	TRUE	TRUE	TRUE	TRUE	FALSE	FALSE	FALSE	FALSE	TRUE
55	Male	Melanoma	Palliative	cICB	CMV-	Validation	TRUE	TRUE	TRUE	TRUE	TRUE	FALSE	FALSE	FALSE	FALSE	FALSE	TRUE
29	Female	Melanoma	Palliative	cICB	CMV-	Discovery	TRUE	TRUE	TRUE	TRUE	TRUE	FALSE	TRUE	FALSE	FALSE	FALSE	FALSE
51	Female	Melanoma	Palliative	cICB	High Titre	Validation	TRUE	TRUE	TRUE	TRUE	TRUE	FALSE	FALSE	FALSE	FALSE	FALSE	FALSE
63	Male	Melanoma	Palliative	cICB	High Titre	Discovery	TRUE	TRUE	TRUE	TRUE	TRUE	FALSE	TRUE	FALSE	FALSE	FALSE	FALSE
64	Male	Melanoma	Palliative	cICB	CMV-	Discovery	TRUE	TRUE	TRUE	TRUE	TRUE	TRUE	FALSE	FALSE	FALSE	FALSE	FALSE
79	Female	Melanoma	Palliative	sICB	High Titre	Discovery	TRUE	TRUE	TRUE	FALSE	TRUE	FALSE	TRUE	FALSE	FALSE	FALSE	FALSE
69	Female	Melanoma	Palliative	sICB	Low Titre	Discovery	TRUE	TRUE	FALSE	FALSE	FALSE	FALSE	FALSE	FALSE	FALSE	FALSE	FALSE
51	Male	Melanoma	Palliative	cICB	CMV-	Discovery	TRUE	TRUE	TRUE	TRUE	TRUE	TRUE	FALSE	TRUE	FALSE	FALSE	FALSE
31	Female	Melanoma	Palliative	cICB	CMV-	Validation	TRUE	TRUE	TRUE	TRUE	TRUE	FALSE	TRUE	FALSE	FALSE	FALSE	TRUE
88	Male	Melanoma	Palliative	sICB	Low Titre	Discovery	TRUE	TRUE	FALSE	FALSE	TRUE	FALSE	FALSE	FALSE	FALSE	FALSE	FALSE
60	Male	Melanoma	Palliative	cICB	CMV-	Discovery	TRUE	TRUE	FALSE	FALSE	TRUE	TRUE	FALSE	FALSE	FALSE	FALSE	FALSE
60	Male	Melanoma	Palliative	cICB	High Titre	Validation	TRUE	TRUE	FALSE	FALSE	TRUE	FALSE	FALSE	FALSE	FALSE	FALSE	FALSE
68	Female	Melanoma	Palliative	cICB	Low Titre	Discovery	TRUE	TRUE	TRUE	TRUE	TRUE	TRUE	FALSE	TRUE	FALSE	TRUE	FALSE
54	Female	Melanoma	Palliative	cICB	Low Titre	Validation	TRUE	TRUE	TRUE	TRUE	TRUE	FALSE	FALSE	FALSE	FALSE	FALSE	FALSE
80	Male	Melanoma	Palliative	sICB	CMV-	Validation	TRUE	TRUE	TRUE	TRUE	TRUE	FALSE	FALSE	FALSE	FALSE	FALSE	FALSE

74	Female	Melanoma	Palliative	cICB	High Titre	Validation	TRUE	TRUE	TRUE	FALSE	TRUE	FALSE	FALSE	FALSE	FALSE	FALSE	FALSE
63	Female	Melanoma	Palliative	cICB	CMV-	Discovery	TRUE	TRUE	TRUE	TRUE	TRUE	FALSE	FALSE	FALSE	FALSE	FALSE	FALSE
71	Male	Melanoma	Palliative	cICB	High Titre	Discovery	TRUE	TRUE	TRUE	TRUE	TRUE	FALSE	FALSE	FALSE	FALSE	FALSE	FALSE
61	Male	Melanoma	Palliative	cICB	CMV-	Validation	TRUE	TRUE	TRUE	TRUE	TRUE	FALSE	FALSE	FALSE	FALSE	FALSE	TRUE
55	Male	Melanoma	Palliative	cICB	CMV-	Validation	TRUE	TRUE	TRUE	TRUE	TRUE	FALSE	FALSE	FALSE	FALSE	FALSE	FALSE
74	Male	Melanoma	Palliative	sICB	High Titre	Discovery	TRUE	TRUE	TRUE	TRUE	TRUE	TRUE	TRUE	TRUE	FALSE	FALSE	FALSE
74	Male	Melanoma	Adjuvant	sICB	Low Titre	Validation	TRUE	FALSE	TRUE	TRUE	TRUE	FALSE	FALSE	FALSE	FALSE	FALSE	FALSE
74	Female	Melanoma	Palliative	cICB	High Titre	Validation	TRUE	TRUE	TRUE	TRUE	TRUE	TRUE	FALSE	TRUE	FALSE	FALSE	FALSE
74	Male	Melanoma	Palliative	cICB	Low Titre	Discovery	TRUE	TRUE	TRUE	TRUE	TRUE	FALSE	FALSE	FALSE	FALSE	FALSE	FALSE
70	Male	Melanoma	Palliative	cICB	CMV-	Validation	TRUE	TRUE	TRUE	TRUE	TRUE	TRUE	FALSE	TRUE	FALSE	FALSE	FALSE
48	Male	Melanoma	Palliative	cICB	CMV-	Discovery	TRUE	TRUE	TRUE	TRUE	TRUE	FALSE	FALSE	FALSE	FALSE	FALSE	TRUE
79	Male	Melanoma	Palliative	sICB	CMV-	Discovery	TRUE	TRUE	TRUE	FALSE	TRUE	TRUE	FALSE	TRUE	FALSE	FALSE	TRUE
68	Male	Melanoma	Palliative	cICB	CMV-	Validation	TRUE	TRUE	FALSE	FALSE	TRUE	FALSE	FALSE	FALSE	FALSE	FALSE	FALSE
75	Male	Melanoma	Palliative	sICB	Low Titre	Validation	TRUE	TRUE	TRUE	TRUE	TRUE	FALSE	FALSE	FALSE	FALSE	FALSE	TRUE
95	Male	Melanoma	Palliative	sICB	Low Titre	Discovery	TRUE	TRUE	TRUE	TRUE	TRUE	TRUE	TRUE	TRUE	FALSE	FALSE	FALSE
56	Female	Melanoma	Palliative	cICB	CMV-	Validation	TRUE	TRUE	FALSE	FALSE	TRUE	TRUE	FALSE	FALSE	FALSE	FALSE	FALSE
60	Male	Melanoma	Palliative	cICB	CMV-	Discovery	TRUE	TRUE	TRUE	TRUE	TRUE	FALSE	TRUE	FALSE	FALSE	FALSE	TRUE
44	Male	Melanoma	Palliative	cICB	CMV-	Discovery	TRUE	TRUE	TRUE	FALSE	TRUE	FALSE	TRUE	FALSE	FALSE	FALSE	FALSE
90	Female	Melanoma	Palliative	sICB	High Titre	Validation	TRUE	TRUE	TRUE	TRUE	TRUE	FALSE	FALSE	FALSE	FALSE	FALSE	FALSE
87	Male	Melanoma	Palliative	sICB	CMV-	Validation	TRUE	TRUE	TRUE	TRUE	TRUE	TRUE	TRUE	TRUE	FALSE	FALSE	FALSE
71	Male	Melanoma	Adjuvant	sICB	CMV-	Discovery	TRUE	FALSE	TRUE	TRUE	TRUE	FALSE	FALSE	FALSE	FALSE	FALSE	FALSE
55	Female	Melanoma	Palliative	cICB	CMV-	Validation	TRUE	TRUE	TRUE	TRUE	TRUE	FALSE	FALSE	FALSE	FALSE	FALSE	FALSE
80	Male	Melanoma	Palliative	sICB	Low Titre	Validation	TRUE	TRUE	TRUE	TRUE	TRUE	TRUE	FALSE	TRUE	TRUE	FALSE	FALSE
34	Female	Melanoma	Palliative	cICB	CMV-	Discovery	TRUE	TRUE	TRUE	TRUE	TRUE	TRUE	TRUE	TRUE	FALSE	FALSE	TRUE
37	Male	Melanoma	Adjuvant	sICB	High Titre	Discovery	TRUE	FALSE	TRUE	TRUE	TRUE	TRUE	TRUE	TRUE	FALSE	FALSE	FALSE
69	Male	Melanoma	Palliative	cICB	CMV-	Discovery	TRUE	TRUE	TRUE	TRUE	TRUE	TRUE	FALSE	TRUE	FALSE	FALSE	FALSE
55	Male	Melanoma	Adjuvant	sICB	High Titre	Validation	TRUE	FALSE	TRUE	TRUE	TRUE	TRUE	TRUE	TRUE	FALSE	FALSE	FALSE
57	Female	Melanoma	Palliative	cICB	CMV-	Validation	TRUE	TRUE	TRUE	TRUE	TRUE	TRUE	FALSE	FALSE	FALSE	FALSE	FALSE
68	Male	Melanoma	Adjuvant	sICB	CMV-	Discovery	TRUE	FALSE	TRUE	TRUE	TRUE	TRUE	FALSE	FALSE	FALSE	FALSE	FALSE
70	Male	Melanoma	Palliative	cICB	CMV-	Validation	TRUE	TRUE	TRUE	TRUE	TRUE	TRUE	FALSE	TRUE	FALSE	FALSE	FALSE

58	Female	Melanoma	Palliative	cICB	High Titre	Validation	TRUE	TRUE	TRUE	TRUE	FALSE	TRUE	TRUE	TRUE	FALSE	FALSE	TRUE
77	Male	Melanoma	Adjuvant	sICB	High Titre	Validation	TRUE	FALSE	FALSE	FALSE	TRUE	FALSE	FALSE	FALSE	FALSE	FALSE	FALSE
81	Male	Melanoma	Palliative	sICB	Low Titre	Discovery	TRUE	TRUE	TRUE	TRUE	FALSE	TRUE	TRUE	TRUE	FALSE	FALSE	TRUE
71	Male	Melanoma	Palliative	cICB	High Titre	Discovery	TRUE	TRUE	TRUE	FALSE	TRUE	FALSE	FALSE	FALSE	FALSE	FALSE	TRUE
72	Female	Melanoma	Palliative	sICB	Low Titre	Discovery	TRUE	TRUE	TRUE	TRUE	TRUE	TRUE	FALSE	TRUE	FALSE	FALSE	FALSE
68	Male	Melanoma	Palliative	cICB	CMV-	Discovery	TRUE	TRUE	TRUE	TRUE	TRUE	TRUE	FALSE	TRUE	FALSE	FALSE	FALSE
58	Female	Melanoma	Palliative	cICB	High Titre	Discovery	TRUE	TRUE	TRUE	TRUE	TRUE	TRUE	FALSE	TRUE	FALSE	FALSE	FALSE
96	Female	Melanoma	Palliative	sICB	High Titre	Validation	TRUE	TRUE	TRUE	TRUE	TRUE	TRUE	FALSE	TRUE	FALSE	FALSE	FALSE
78	Female	Melanoma	Palliative	sICB	CMV-	Validation	TRUE	TRUE	TRUE	TRUE	FALSE	TRUE	TRUE	TRUE	FALSE	FALSE	TRUE
83	Female	Melanoma	Palliative	sICB	CMV-	Validation	TRUE	TRUE	TRUE	TRUE	TRUE	TRUE	FALSE	TRUE	FALSE	FALSE	TRUE
70	Female	Melanoma	Adjuvant	sICB	CMV-	Discovery	TRUE	FALSE	FALSE	FALSE	TRUE	FALSE	FALSE	FALSE	FALSE	FALSE	FALSE
60	Male	Melanoma	Palliative	cICB	Low Titre	Discovery	TRUE	TRUE	TRUE	FALSE	TRUE	TRUE	FALSE	TRUE	FALSE	FALSE	TRUE
40	Male	Melanoma	Palliative	cICB	High Titre	Discovery	TRUE	TRUE	FALSE	FALSE	TRUE	FALSE	FALSE	FALSE	FALSE	FALSE	FALSE
68	Female	Melanoma	Palliative	cICB	Low Titre	Validation	TRUE	TRUE	TRUE	TRUE	TRUE	FALSE	FALSE	FALSE	FALSE	FALSE	FALSE
47	Male	Melanoma	Palliative	cICB	High Titre	Validation	TRUE	TRUE	TRUE	TRUE	FALSE	FALSE	TRUE	FALSE	FALSE	FALSE	FALSE
51	Male	Melanoma	Palliative	cICB	CMV-	Validation	TRUE	TRUE	TRUE	TRUE	TRUE	TRUE	FALSE	TRUE	FALSE	FALSE	FALSE
44	Male	Melanoma	Palliative	cICB	High Titre	Discovery	TRUE	TRUE	TRUE	TRUE	TRUE	FALSE	FALSE	FALSE	FALSE	FALSE	FALSE
58	Female	RCC	Palliative	cICB	Low Titre	Discovery	TRUE	TRUE	TRUE	TRUE	TRUE	FALSE	FALSE	FALSE	FALSE	FALSE	FALSE
73	Female	Melanoma	Palliative	cICB	CMV-	Validation	TRUE	TRUE	TRUE	TRUE	TRUE	TRUE	TRUE	TRUE	FALSE	FALSE	FALSE
85	Male	Melanoma	Palliative	sICB	CMV-	Discovery	TRUE	TRUE	TRUE	FALSE	TRUE	TRUE	FALSE	TRUE	FALSE	FALSE	FALSE
32	Female	Melanoma	Palliative	cICB	CMV-	Discovery	TRUE	TRUE	TRUE	TRUE	TRUE	TRUE	FALSE	TRUE	FALSE	FALSE	FALSE
81	Male	Melanoma	Palliative	sICB	CMV-	Discovery	TRUE	TRUE	TRUE	FALSE	TRUE	TRUE	TRUE	TRUE	FALSE	FALSE	TRUE
60	Male	Melanoma	Palliative	cICB	Low Titre	Validation	TRUE	TRUE	TRUE	TRUE	TRUE	TRUE	FALSE	TRUE	FALSE	FALSE	TRUE
68	Male	Melanoma	Palliative	cICB	Low Titre	Validation	TRUE	TRUE	TRUE	TRUE	TRUE	TRUE	TRUE	TRUE	FALSE	TRUE	FALSE
62	Male	RCC	Palliative	cICB	High Titre	Discovery	TRUE	TRUE	TRUE	TRUE	TRUE	FALSE	FALSE	FALSE	FALSE	FALSE	FALSE
47	Male	Melanoma	Palliative	cICB	High Titre	Validation	TRUE	TRUE	TRUE	TRUE	TRUE	TRUE	FALSE	TRUE	FALSE	FALSE	FALSE
80	Male	Melanoma	Palliative	sICB	Low Titre	Discovery	TRUE	TRUE	FALSE	FALSE	TRUE	TRUE	FALSE	TRUE	FALSE	FALSE	FALSE
79	Male	Melanoma	Palliative	sICB	CMV-	Validation	TRUE	TRUE	FALSE	FALSE	TRUE	TRUE	FALSE	TRUE	FALSE	FALSE	FALSE
71	Female	Melanoma	Palliative	cICB	CMV-	Validation	TRUE	TRUE	TRUE	TRUE	FALSE	FALSE	TRUE	FALSE	FALSE	FALSE	FALSE
57	Male	Melanoma	Palliative	cICB	CMV-	Validation	TRUE	TRUE	TRUE	FALSE	TRUE	TRUE	FALSE	TRUE	FALSE	FALSE	FALSE

47	Male	Melanoma	Palliative	cICB	LowTitre	Validation	TRUE	TRUE	TRUE	TRUE	TRUE	TRUE	TRUE	TRUE	FALSE	FALSE	TRUE
57	Male	RCC	Palliative	cICB	CMV-	Discovery	TRUE	TRUE	TRUE	TRUE	TRUE	TRUE	FALSE	FALSE	FALSE	FALSE	FALSE
74	Male	Melanoma	Adjuvant	sICB	HighTitre	Discovery	TRUE	FALSE	TRUE	TRUE	TRUE	FALSE	FALSE	FALSE	FALSE	FALSE	FALSE
70	Male	RCC	Palliative	cICB	CMV-	Validation	TRUE	TRUE	TRUE	TRUE	TRUE	TRUE	FALSE	FALSE	FALSE	FALSE	FALSE
53	Male	RCC	Palliative	cICB	CMV-	Discovery	TRUE	TRUE	TRUE	TRUE	TRUE	FALSE	FALSE	FALSE	FALSE	FALSE	FALSE
71	Male	RCC	Palliative	cICB	CMV-	Discovery	TRUE	TRUE	TRUE	TRUE	TRUE	FALSE	FALSE	FALSE	FALSE	FALSE	FALSE
63	Female	Melanoma	Palliative	cICB	LowTitre	Validation	TRUE	TRUE	TRUE	TRUE	TRUE	TRUE	FALSE	TRUE	FALSE	FALSE	FALSE
59	Male	Melanoma	Palliative	cICB	CMV-	Validation	TRUE	TRUE	TRUE	TRUE	TRUE	FALSE	FALSE	FALSE	FALSE	FALSE	FALSE
47	Female	Melanoma	Palliative	cICB	CMV-	Discovery	TRUE	TRUE	TRUE	FALSE	TRUE	TRUE	FALSE	TRUE	FALSE	FALSE	FALSE
51	Female	Melanoma	Palliative	cICB	LowTitre	Discovery	TRUE	TRUE	TRUE	FALSE	TRUE	TRUE	FALSE	TRUE	FALSE	FALSE	TRUE
65	Female	Melanoma	Adjuvant	sICB	HighTitre	Validation	TRUE	FALSE	TRUE	TRUE	TRUE	FALSE	FALSE	FALSE	FALSE	FALSE	FALSE
36	Female	Melanoma	Palliative	cICB	CMV-	Validation	TRUE	TRUE	TRUE	FALSE	TRUE	TRUE	FALSE	TRUE	FALSE	FALSE	TRUE
70	Female	Melanoma	Palliative	cICB	CMV-	Discovery	TRUE	TRUE	TRUE	FALSE	TRUE	TRUE	FALSE	TRUE	FALSE	FALSE	FALSE
72	Male	Melanoma	Palliative	cICB	HighTitre	Validation	TRUE	TRUE	TRUE	TRUE	TRUE	FALSE	FALSE	FALSE	FALSE	FALSE	FALSE
48	Female	Melanoma	Adjuvant	sICB	LowTitre	Discovery	TRUE	FALSE	TRUE	TRUE	TRUE	FALSE	FALSE	FALSE	FALSE	FALSE	FALSE
54	Male	Melanoma	Palliative	cICB	CMV-	Discovery	TRUE	TRUE	TRUE	TRUE	TRUE	TRUE	TRUE	TRUE	FALSE	FALSE	FALSE
74	Female	Melanoma	Palliative	cICB	LowTitre	Discovery	TRUE	TRUE	FALSE	FALSE	TRUE	TRUE	FALSE	TRUE	FALSE	FALSE	FALSE
48	Female	Melanoma	Palliative	cICB	HighTitre	Discovery	TRUE	TRUE	TRUE	FALSE	TRUE	TRUE	TRUE	TRUE	FALSE	FALSE	FALSE
59	Male	Melanoma	Palliative	cICB	CMV-	Discovery	TRUE	TRUE	TRUE	TRUE	TRUE	TRUE	FALSE	TRUE	FALSE	FALSE	FALSE
69	Male	RCC	Palliative	cICB	HighTitre	Discovery	TRUE	TRUE	FALSE	FALSE	TRUE	FALSE	FALSE	FALSE	TRUE	FALSE	FALSE
62	Female	Melanoma	Palliative	cICB	CMV-	Discovery	TRUE	TRUE	TRUE	TRUE	TRUE	TRUE	TRUE	TRUE	FALSE	FALSE	TRUE
69	Female	RCC	Palliative	cICB	CMV-	Discovery	TRUE	TRUE	TRUE	TRUE	TRUE	TRUE	FALSE	TRUE	FALSE	FALSE	FALSE
29	Male	Melanoma	Palliative	cICB	CMV-	Validation	TRUE	TRUE	TRUE	TRUE	TRUE	TRUE	FALSE	TRUE	FALSE	FALSE	FALSE
69	Male	Melanoma	Palliative	cICB	CMV-	Discovery	TRUE	TRUE	TRUE	TRUE	TRUE	FALSE	FALSE	TRUE	FALSE	FALSE	FALSE
73	Male	Melanoma	Palliative	cICB	LowTitre	Validation	TRUE	TRUE	TRUE	FALSE	TRUE	FALSE	FALSE	TRUE	FALSE	FALSE	FALSE
54	Female	RCC	Palliative	cICB	CMV-	Discovery	TRUE	TRUE	TRUE	TRUE	TRUE	FALSE	FALSE	FALSE	FALSE	FALSE	FALSE
57	Male	Melanoma	Palliative	cICB	CMV-	Validation	TRUE	TRUE	TRUE	FALSE	TRUE	TRUE	FALSE	TRUE	FALSE	FALSE	TRUE
50	Female	Melanoma	Palliative	cICB	HighTitre	Discovery	TRUE	TRUE	TRUE	TRUE	TRUE	FALSE	FALSE	FALSE	FALSE	FALSE	FALSE
70	Male	Melanoma	Palliative	cICB	HighTitre	Validation	TRUE	TRUE	TRUE	TRUE	TRUE	FALSE	FALSE	FALSE	TRUE	FALSE	FALSE
69	Male	RCC	Palliative	cICB	CMV-	Discovery	TRUE	TRUE	TRUE	TRUE	TRUE	FALSE	FALSE	FALSE	FALSE	FALSE	FALSE

72	Male	Melanoma	Palliative	cICB	CMV-	Discovery	TRUE	TRUE	FALSE	FALSE	TRUE	FALSE	FALSE	FALSE	FALSE	FALSE	FALSE
71	Female	Melanoma	Palliative	cICB	CMV-	Discovery	TRUE	TRUE	TRUE	TRUE	TRUE	FALSE	TRUE	FALSE	FALSE	FALSE	TRUE
64	Male	RCC	Palliative	cICB	Low Titre	Discovery	TRUE	TRUE	TRUE	TRUE	TRUE	FALSE	FALSE	FALSE	FALSE	FALSE	FALSE
52	Male	RCC	Palliative	cICB	CMV-	Discovery	TRUE	TRUE	TRUE	TRUE	TRUE	FALSE	FALSE	FALSE	FALSE	FALSE	FALSE
61	Female	Melanoma	Palliative	cICB	CMV-	Validation	TRUE	TRUE	TRUE	TRUE	TRUE	FALSE	FALSE	FALSE	FALSE	FALSE	FALSE
57	Male	RCC	Palliative	cICB	CMV-	Validation	TRUE	TRUE	TRUE	TRUE	TRUE	FALSE	FALSE	FALSE	FALSE	FALSE	FALSE
67	Male	Melanoma	Palliative	cICB	CMV-	Validation	TRUE	TRUE	TRUE	TRUE	TRUE	FALSE	FALSE	FALSE	FALSE	FALSE	TRUE
46	Female	Melanoma	Palliative	cICB	CMV-	Discovery	TRUE	TRUE	TRUE	TRUE	TRUE	FALSE	FALSE	FALSE	FALSE	FALSE	FALSE
69	Female	Melanoma	Adjuvant	sICB	High Titre	Validation	TRUE	FALSE	TRUE	TRUE	TRUE	FALSE	FALSE	FALSE	FALSE	FALSE	FALSE
81	Male	Melanoma	Palliative	sICB	CMV-	Validation	TRUE	TRUE	TRUE	TRUE	TRUE	FALSE	FALSE	FALSE	FALSE	FALSE	FALSE
63	Male	Melanoma	Palliative	cICB	CMV-	Validation	TRUE	TRUE	TRUE	TRUE	TRUE	FALSE	FALSE	FALSE	FALSE	FALSE	FALSE
68	Male	Melanoma	Palliative	cICB	CMV-	Validation	TRUE	TRUE	TRUE	TRUE	FALSE	FALSE	FALSE	FALSE	FALSE	FALSE	FALSE
51	Female	Melanoma	Palliative	cICB	CMV-	Discovery	TRUE	TRUE	TRUE	TRUE	TRUE	FALSE	FALSE	FALSE	FALSE	FALSE	FALSE
73	Female	Melanoma	Palliative	cICB	Low Titre	Discovery	TRUE	TRUE	TRUE	TRUE	TRUE	FALSE	FALSE	FALSE	FALSE	FALSE	FALSE
74	Male	RCC	Palliative	cICB	High Titre	Discovery	TRUE	TRUE	TRUE	TRUE	TRUE	FALSE	FALSE	FALSE	TRUE	FALSE	FALSE
64	Male	Melanoma	Palliative	cICB	CMV-	Discovery	TRUE	TRUE	TRUE	TRUE	TRUE	FALSE	FALSE	FALSE	FALSE	FALSE	FALSE
55	Male	Melanoma	Palliative	cICB	CMV-	Discovery	TRUE	TRUE	TRUE	TRUE	TRUE	FALSE	FALSE	FALSE	FALSE	FALSE	FALSE
47	Female	Melanoma	Palliative	cICB	High Titre	Validation	TRUE	TRUE	TRUE	TRUE	TRUE	FALSE	FALSE	FALSE	FALSE	FALSE	FALSE
80	Male	Melanoma	Palliative	cICB	CMV-	Validation	TRUE	TRUE	TRUE	TRUE	TRUE	FALSE	FALSE	FALSE	FALSE	FALSE	FALSE
61	Male	Melanoma	Palliative	cICB	CMV-	Discovery	TRUE	TRUE	FALSE	FALSE	TRUE	FALSE	FALSE	FALSE	FALSE	FALSE	FALSE
63	Male	Melanoma	Palliative	cICB	Low Titre	Discovery	TRUE	TRUE	TRUE	TRUE	FALSE	FALSE	FALSE	FALSE	FALSE	FALSE	FALSE
32	Male	Melanoma	Palliative	cICB	CMV-	Discovery	TRUE	TRUE	TRUE	TRUE	TRUE	FALSE	FALSE	FALSE	FALSE	FALSE	FALSE
70	Male	Melanoma	Palliative	cICB	Low Titre	Validation	TRUE	TRUE	TRUE	TRUE	TRUE	FALSE	FALSE	FALSE	TRUE	FALSE	FALSE
33	Male	Melanoma	Palliative	cICB	CMV-	Discovery	TRUE	TRUE	TRUE	TRUE	FALSE	FALSE	FALSE	FALSE	FALSE	FALSE	FALSE
56	Male	RCC	Palliative	cICB	CMV-	Validation	TRUE	TRUE	TRUE	TRUE	TRUE	FALSE	FALSE	FALSE	FALSE	FALSE	FALSE
84	Male	RCC	Palliative	cICB	CMV-	Discovery	TRUE	TRUE	TRUE	TRUE	TRUE	FALSE	FALSE	FALSE	FALSE	FALSE	FALSE
58	Female	Melanoma	Palliative	cICB	High Titre	Validation	TRUE	TRUE	TRUE	TRUE	FALSE	FALSE	FALSE	FALSE	FALSE	FALSE	FALSE
81	Female	Melanoma	Palliative	sICB	CMV-	Discovery	TRUE	TRUE	TRUE	TRUE	TRUE	FALSE	FALSE	FALSE	FALSE	FALSE	FALSE
82	Female	Melanoma	Palliative	sICB	Low Titre	Discovery	TRUE	TRUE	TRUE	TRUE	FALSE	FALSE	FALSE	FALSE	FALSE	FALSE	FALSE
72	Male	Melanoma	Adjuvant	sICB	Low Titre	Discovery	TRUE	FALSE	TRUE	TRUE	FALSE	FALSE	FALSE	FALSE	TRUE	FALSE	FALSE

63	Female	Melanoma	Palliative	cICB	CMV-	Validation	TRUE	TRUE	TRUE	TRUE	FALSE	FALSE	FALSE	FALSE	FALSE	FALSE	FALSE
42	Female	Melanoma	Palliative	cICB	Low Titre	Validation	TRUE	TRUE	TRUE	TRUE	TRUE	FALSE	FALSE	FALSE	FALSE	FALSE	FALSE
64	Female	Melanoma	Palliative	cICB	CMV-	Discovery	TRUE	TRUE	TRUE	TRUE	TRUE	FALSE	FALSE	FALSE	FALSE	FALSE	FALSE
93	Male	cSCC	Palliative	sICB	High Titre	Validation	TRUE	TRUE	FALSE	FALSE	FALSE	FALSE	FALSE	FALSE	FALSE	FALSE	FALSE
40	Male	Melanoma	Palliative	cICB	Low Titre	Discovery	TRUE	TRUE	TRUE	TRUE	TRUE	FALSE	FALSE	FALSE	FALSE	FALSE	FALSE
77	Male	Melanoma	Adjuvant	sICB	High Titre	Discovery	TRUE	FALSE	TRUE	TRUE	TRUE	FALSE	FALSE	FALSE	FALSE	FALSE	FALSE
76	Male	Melanoma	Adjuvant	sICB	CMV-	Discovery	TRUE	FALSE	TRUE	TRUE	TRUE	FALSE	FALSE	FALSE	FALSE	FALSE	FALSE
43	Female	Melanoma	Palliative	cICB	High Titre	Validation	TRUE	TRUE	TRUE	TRUE	TRUE	FALSE	FALSE	FALSE	TRUE	FALSE	FALSE
60	Male	Melanoma	Palliative	cICB	High Titre	Discovery	TRUE	TRUE	TRUE	TRUE	TRUE	FALSE	FALSE	FALSE	FALSE	FALSE	FALSE
65	Female	Melanoma	Palliative	cICB	CMV-	Validation	TRUE	TRUE	TRUE	TRUE	TRUE	FALSE	FALSE	FALSE	FALSE	FALSE	FALSE
57	Female	Melanoma	Adjuvant	sICB	CMV-	Discovery	TRUE	FALSE	TRUE	TRUE	TRUE	FALSE	FALSE	FALSE	FALSE	FALSE	FALSE
84	Female	Melanoma	Palliative	sICB	High Titre	Discovery	TRUE	TRUE	TRUE	TRUE	FALSE	FALSE	FALSE	FALSE	FALSE	FALSE	FALSE
76	Male	Mesothelic	Palliative	sICB	CMV-	Validation	TRUE	TRUE	TRUE	TRUE	FALSE	FALSE	FALSE	FALSE	FALSE	FALSE	FALSE
52	Male	Colon	Palliative	sICB	High Titre	Discovery	TRUE	TRUE	TRUE	TRUE	FALSE	FALSE	FALSE	FALSE	FALSE	FALSE	FALSE
76	Male	Melanoma	Palliative	sICB	Low Titre	Validation	TRUE	TRUE	TRUE	TRUE	TRUE	FALSE	FALSE	FALSE	FALSE	FALSE	FALSE
75	Male	RCC	Palliative	cICB	Low Titre	Discovery	TRUE	TRUE	TRUE	TRUE	TRUE	FALSE	FALSE	FALSE	FALSE	FALSE	FALSE
50	Female	Melanoma	Palliative	cICB	CMV-	Validation	TRUE	TRUE	TRUE	FALSE	TRUE	FALSE	FALSE	FALSE	FALSE	FALSE	FALSE
79	Male	Melanoma	Palliative	sICB	CMV-	Validation	TRUE	TRUE	TRUE	TRUE	FALSE	FALSE	FALSE	FALSE	FALSE	FALSE	FALSE
73	Female	Melanoma	Palliative	cICB	High Titre	Discovery	TRUE	TRUE	TRUE	TRUE	TRUE	FALSE	FALSE	FALSE	TRUE	FALSE	FALSE
48	Female	Melanoma	Adjuvant	sICB	High Titre	Discovery	TRUE	FALSE	TRUE	TRUE	TRUE	TRUE	TRUE	TRUE	FALSE	FALSE	FALSE
53	Female	Melanoma	Palliative	cICB	CMV-	Discovery	TRUE	TRUE	TRUE	TRUE	TRUE	FALSE	FALSE	FALSE	FALSE	FALSE	FALSE
68	Female	Melanoma	Palliative	cICB	CMV-	Discovery	TRUE	TRUE	TRUE	FALSE	TRUE	FALSE	FALSE	FALSE	FALSE	FALSE	FALSE
52	Female	Melanoma	Adjuvant	sICB	Low Titre	Discovery	TRUE	FALSE	TRUE	TRUE	TRUE	FALSE	FALSE	FALSE	TRUE	FALSE	FALSE
18	Female	Melanoma	Palliative	cICB	CMV-	Validation	TRUE	TRUE	TRUE	TRUE	TRUE	FALSE	FALSE	FALSE	FALSE	FALSE	FALSE
67	Female	Colon	Palliative	sICB	CMV-	Validation	TRUE	TRUE	TRUE	TRUE	FALSE	FALSE	FALSE	FALSE	FALSE	FALSE	FALSE
74	Female	Colon	Palliative	sICB	Low Titre	Discovery	TRUE	TRUE	TRUE	TRUE	FALSE	FALSE	FALSE	FALSE	TRUE	FALSE	FALSE
63	Male	Melanoma	Palliative	cICB	CMV-	Validation	TRUE	TRUE	TRUE	FALSE	TRUE	FALSE	FALSE	FALSE	FALSE	FALSE	FALSE
83	Female	Melanoma	Palliative	sICB	High Titre	Validation	TRUE	TRUE	TRUE	TRUE	TRUE	FALSE	FALSE	FALSE	FALSE	FALSE	FALSE
68	Male	Melanoma	Palliative	cICB	High Titre	Discovery	TRUE	TRUE	TRUE	TRUE	TRUE	FALSE	FALSE	FALSE	FALSE	FALSE	FALSE
54	Male	Melanoma	Palliative	cICB	CMV-	Validation	TRUE	TRUE	FALSE	FALSE	TRUE	FALSE	FALSE	FALSE	FALSE	FALSE	FALSE

84	Female	Colon	Palliative	sICB	High Titre	Discovery	TRUE	TRUE	TRUE	TRUE	FALSE	FALSE	FALSE	FALSE	FALSE	FALSE	FALSE
43	Male	Melanoma	Palliative	cICB	CMV-	Validation	TRUE	TRUE	TRUE	TRUE	TRUE	FALSE	FALSE	FALSE	FALSE	FALSE	FALSE
75	Male	Melanoma	Adjuvant	sICB	CMV-	Validation	TRUE	FALSE	TRUE	TRUE	TRUE	FALSE	FALSE	FALSE	FALSE	FALSE	FALSE
56	Male	Melanoma	Palliative	cICB	CMV-	Discovery	TRUE	TRUE	FALSE	FALSE	TRUE	FALSE	FALSE	FALSE	FALSE	FALSE	FALSE
64	Male	Melanoma	Adjuvant	sICB	CMV-	Discovery	TRUE	FALSE	TRUE	TRUE	TRUE	FALSE	FALSE	FALSE	FALSE	FALSE	FALSE
36	Female	Melanoma	Adjuvant	sICB	CMV-	Discovery	TRUE	FALSE	TRUE	TRUE	TRUE	FALSE	FALSE	FALSE	FALSE	FALSE	FALSE
64	Male	Melanoma	Palliative	cICB	CMV-	Validation	TRUE	TRUE	TRUE	FALSE	TRUE	FALSE	FALSE	FALSE	FALSE	FALSE	FALSE
67	Male	Melanoma	Adjuvant	sICB	Low Titre	Discovery	TRUE	FALSE	TRUE	TRUE	TRUE	TRUE	TRUE	FALSE	FALSE	FALSE	FALSE
63	Female	Melanoma	Palliative	cICB	CMV-	Validation	TRUE	TRUE	TRUE	TRUE	FALSE	FALSE	FALSE	FALSE	FALSE	FALSE	FALSE
71	Female	Colon	Palliative	sICB	High Titre	Validation	TRUE	TRUE	TRUE	TRUE	FALSE	FALSE	FALSE	FALSE	FALSE	FALSE	FALSE
73	Female	Melanoma	Palliative	cICB	Low Titre	Validation	TRUE	TRUE	TRUE	TRUE	TRUE	TRUE	FALSE	FALSE	FALSE	FALSE	FALSE
73	Male	Melanoma	Palliative	cICB	CMV-	Validation	TRUE	TRUE	TRUE	TRUE	FALSE	FALSE	FALSE	FALSE	FALSE	FALSE	FALSE
85	Female	Melanoma	Palliative	sICB	Low Titre	Validation	TRUE	TRUE	TRUE	TRUE	TRUE	FALSE	FALSE	FALSE	FALSE	FALSE	FALSE
76	Male	Melanoma	Palliative	cICB	CMV-	Validation	TRUE	TRUE	FALSE	FALSE	TRUE	FALSE	FALSE	FALSE	FALSE	FALSE	FALSE
62	Male	Melanoma	Palliative	cICB	CMV-	Discovery	TRUE	TRUE	TRUE	TRUE	FALSE	TRUE	TRUE	FALSE	FALSE	FALSE	FALSE
67	Female	Melanoma	Adjuvant	sICB	Low Titre	Validation	TRUE	FALSE	TRUE	TRUE	TRUE	TRUE	FALSE	FALSE	TRUE	FALSE	FALSE
66	Female	Melanoma	Palliative	cICB	Low Titre	Discovery	TRUE	TRUE	TRUE	TRUE	FALSE	TRUE	FALSE	FALSE	FALSE	FALSE	FALSE
80	Male	Melanoma	Palliative	sICB	Low Titre	Validation	TRUE	TRUE	TRUE	TRUE	FALSE	FALSE	FALSE	FALSE	FALSE	FALSE	FALSE
76	Male	Melanoma	Palliative	cICB	Low Titre	Discovery	TRUE	TRUE	TRUE	TRUE	TRUE	FALSE	FALSE	FALSE	FALSE	FALSE	FALSE
41	Male	RCC	Palliative	cICB	CMV-	Validation	TRUE	TRUE	TRUE	TRUE	FALSE	FALSE	FALSE	FALSE	FALSE	FALSE	FALSE
84	Male	Colon	Palliative	sICB	CMV-	Validation	TRUE	TRUE	TRUE	TRUE	FALSE	FALSE	FALSE	FALSE	FALSE	FALSE	FALSE
74	Female	Melanoma	Adjuvant	sICB	High Titre	Discovery	TRUE	FALSE	TRUE	TRUE	FALSE	FALSE	FALSE	FALSE	FALSE	FALSE	FALSE
38	Male	RCC	Palliative	cICB	High Titre	Discovery	TRUE	TRUE	TRUE	TRUE	TRUE	FALSE	FALSE	FALSE	FALSE	FALSE	FALSE
53	Male	Melanoma	Palliative	cICB	Low Titre	Discovery	TRUE	TRUE	TRUE	TRUE	TRUE	TRUE	TRUE	TRUE	FALSE	FALSE	FALSE
84	Male	cSCC	Palliative	sICB	CMV-	Discovery	TRUE	TRUE	FALSE	FALSE	TRUE	TRUE	FALSE	FALSE	FALSE	FALSE	FALSE
79	Male	Melanoma	Palliative	cICB	High Titre	Discovery	TRUE	TRUE	FALSE	FALSE	FALSE	FALSE	FALSE	FALSE	TRUE	FALSE	FALSE
49	Female	Melanoma	Palliative	cICB	Low Titre	Discovery	TRUE	TRUE	TRUE	TRUE	FALSE	FALSE	FALSE	FALSE	FALSE	FALSE	FALSE
57	Female	Melanoma	Palliative	cICB	CMV-	Discovery	TRUE	TRUE	TRUE	TRUE	FALSE	FALSE	FALSE	FALSE	FALSE	FALSE	FALSE
39	Male	Melanoma	Palliative	cICB	CMV-	Validation	TRUE	TRUE	TRUE	TRUE	FALSE	FALSE	FALSE	FALSE	FALSE	FALSE	FALSE
41	Male	Melanoma	Palliative	cICB	CMV-	Validation	TRUE	TRUE	TRUE	TRUE	FALSE	FALSE	FALSE	FALSE	FALSE	FALSE	FALSE

34	Male	Melanoma	Adjuvant	sICB	LowTitre	Validation	TRUE	FALSE	TRUE	TRUE	FALSE	TRUE	FALSE	TRUE	FALSE	FALSE	FALSE
69	Female	Melanoma	Palliative	cICB	CMV-	Discovery	TRUE	TRUE	TRUE	TRUE	FALSE	FALSE	FALSE	FALSE	FALSE	FALSE	FALSE
29	Male	Melanoma	Palliative	cICB	LowTitre	Discovery	TRUE	TRUE	TRUE	TRUE	FALSE	FALSE	FALSE	FALSE	TRUE	FALSE	FALSE
44	Female	Colon	Palliative	sICB	HighTitre	Discovery	TRUE	TRUE	TRUE	TRUE	FALSE	FALSE	FALSE	FALSE	TRUE	FALSE	FALSE
76	Male	Melanoma	Palliative	sICB	HighTitre	Validation	TRUE	TRUE	TRUE	TRUE	FALSE	FALSE	FALSE	FALSE	FALSE	FALSE	FALSE
47	Female	Melanoma	Adjuvant	sICB	LowTitre	Validation	TRUE	FALSE	TRUE	TRUE	FALSE	FALSE	FALSE	FALSE	FALSE	FALSE	FALSE
54	Male	Melanoma	Palliative	cICB	LowTitre	Validation	TRUE	TRUE	TRUE	TRUE	FALSE	TRUE	TRUE	FALSE	FALSE	FALSE	FALSE
75	Male	Melanoma	Palliative	cICB	LowTitre	Validation	TRUE	TRUE	TRUE	TRUE	FALSE	TRUE	FALSE	FALSE	FALSE	FALSE	FALSE
68	Male	cSCC	Palliative	sICB	HighTitre	Discovery	TRUE	TRUE	TRUE	TRUE	FALSE	TRUE	TRUE	TRUE	FALSE	FALSE	FALSE
66	Male	Melanoma	Palliative	cICB	CMV-	Discovery	TRUE	TRUE	TRUE	TRUE	FALSE	FALSE	FALSE	FALSE	FALSE	FALSE	FALSE
23	Male	Melanoma	Palliative	cICB	CMV-	Validation	TRUE	TRUE	TRUE	TRUE	FALSE	FALSE	FALSE	FALSE	FALSE	FALSE	FALSE
54	Male	Melanoma	Palliative	cICB	LowTitre	Validation	TRUE	TRUE	TRUE	TRUE	FALSE	TRUE	FALSE	TRUE	FALSE	FALSE	FALSE
81	Female	Melanoma	Palliative	sICB	LowTitre	Discovery	TRUE	TRUE	FALSE	FALSE	FALSE	FALSE	FALSE	FALSE	FALSE	FALSE	FALSE
38	Female	Melanoma	Palliative	cICB	HighTitre	Discovery	TRUE	TRUE	FALSE	FALSE	FALSE	TRUE	FALSE	TRUE	FALSE	FALSE	FALSE
72	Female	Melanoma	Palliative	cICB	CMV-	Validation	TRUE	TRUE	TRUE	TRUE	FALSE	FALSE	FALSE	FALSE	FALSE	FALSE	FALSE
76	Male	Melanoma	Palliative	cICB	LowTitre	Discovery	TRUE	TRUE	FALSE	FALSE	FALSE	FALSE	FALSE	FALSE	FALSE	FALSE	FALSE
77	Female	Melanoma	Palliative	cICB	HighTitre	Discovery	TRUE	TRUE	TRUE	TRUE	FALSE	FALSE	FALSE	FALSE	TRUE	FALSE	FALSE
80	Male	Melanoma	Palliative	cICB	LowTitre	Discovery	TRUE	TRUE	TRUE	FALSE	FALSE	TRUE	FALSE	TRUE	FALSE	FALSE	FALSE
65	Female	Melanoma	Adjuvant	sICB	HighTitre	Validation	TRUE	FALSE	FALSE	FALSE	FALSE	TRUE	FALSE	TRUE	TRUE	FALSE	FALSE
79	Female	Melanoma	Palliative	cICB	CMV-	Validation	TRUE	TRUE	FALSE	FALSE	FALSE	TRUE	FALSE	TRUE	FALSE	FALSE	FALSE
66	Female	Melanoma	Palliative	cICB	LowTitre	Discovery	TRUE	TRUE	TRUE	TRUE	FALSE	TRUE	TRUE	TRUE	FALSE	FALSE	FALSE
80	Female	Melanoma	Palliative	sICB	LowTitre	Discovery	TRUE	TRUE	TRUE	TRUE	FALSE	TRUE	FALSE	TRUE	FALSE	FALSE	FALSE
49	Female	Melanoma	Palliative	cICB	LowTitre	Discovery	TRUE	TRUE	TRUE	TRUE	FALSE	FALSE	FALSE	FALSE	FALSE	FALSE	FALSE
77	Female	Melanoma	Palliative	cICB	HighTitre	Discovery	TRUE	TRUE	TRUE	TRUE	FALSE	TRUE	TRUE	FALSE	FALSE	FALSE	FALSE
67	Male	RCC	Adjuvant	sICB	LowTitre	Discovery	TRUE	FALSE	TRUE	TRUE	TRUE	FALSE	FALSE	FALSE	FALSE	FALSE	FALSE
75	Male	Melanoma	Palliative	cICB	CMV-	Validation	TRUE	TRUE	FALSE	FALSE	TRUE	TRUE	FALSE	TRUE	FALSE	FALSE	FALSE
61	Male	Colon	Palliative	cICB	LowTitre	Discovery	TRUE	TRUE	TRUE	TRUE	FALSE	FALSE	FALSE	FALSE	FALSE	FALSE	FALSE
80	Male	Mesothelic	Palliative	cICB	CMV-	Validation	TRUE	TRUE	TRUE	TRUE	FALSE	FALSE	FALSE	FALSE	FALSE	FALSE	FALSE
54	Female	Colon	Palliative	sICB	CMV-	Validation	TRUE	TRUE	TRUE	TRUE	FALSE	FALSE	FALSE	FALSE	FALSE	FALSE	FALSE
72	Male	Mesothelic	Palliative	cICB	HighTitre	Validation	TRUE	TRUE	TRUE	TRUE	FALSE	FALSE	FALSE	FALSE	TRUE	FALSE	FALSE

66	Female	RCC	Adjuvant	sICB	Low Titre	Discovery	TRUE	FALSE	FALSE	FALSE	TRUE	TRUE	FALSE	FALSE	FALSE	FALSE	FALSE
77	Male	cSCC	Palliative	sICB	CMV-	Discovery	TRUE	TRUE	TRUE	TRUE	FALSE	FALSE	FALSE	FALSE	FALSE	FALSE	FALSE
72	Male	Melanoma	Palliative	cICB	CMV-	Discovery	TRUE	TRUE	TRUE	TRUE	TRUE	TRUE	FALSE	TRUE	FALSE	FALSE	FALSE
84	Male	Melanoma	Palliative	sICB	Low Titre	Validation	TRUE	TRUE	TRUE	TRUE	TRUE	TRUE	FALSE	TRUE	FALSE	FALSE	FALSE
65	Male	Melanoma	Palliative	cICB	CMV-	Validation	TRUE	TRUE	TRUE	TRUE	FALSE	TRUE	FALSE	TRUE	FALSE	FALSE	FALSE
44	Male	Melanoma	Palliative	cICB	CMV-	Validation	TRUE	TRUE	TRUE	TRUE	FALSE	FALSE	FALSE	FALSE	FALSE	FALSE	FALSE
70	Male	Colon	Palliative	sICB	CMV-	Validation	TRUE	TRUE	TRUE	TRUE	FALSE	FALSE	FALSE	FALSE	FALSE	FALSE	FALSE
56	Male	Melanoma	Palliative	cICB	Low Titre	Discovery	TRUE	TRUE	TRUE	TRUE	FALSE	TRUE	TRUE	TRUE	FALSE	FALSE	FALSE
53	Male	Melanoma	Palliative	cICB	Low Titre	Discovery	TRUE	TRUE	TRUE	TRUE	FALSE	TRUE	FALSE	TRUE	TRUE	FALSE	FALSE
78	Female	Melanoma	Palliative	cICB	Low Titre	Validation	TRUE	TRUE	TRUE	TRUE	FALSE	FALSE	FALSE	FALSE	FALSE	FALSE	FALSE
73	Male	Melanoma	Palliative	cICB	CMV-	Discovery	TRUE	TRUE	TRUE	TRUE	FALSE	TRUE	FALSE	TRUE	FALSE	FALSE	FALSE
82	Female	Melanoma	Palliative	sICB	High Titre	Discovery	TRUE	TRUE	TRUE	TRUE	FALSE	FALSE	FALSE	FALSE	FALSE	FALSE	FALSE
78	Male	Mesothelic	Palliative	cICB	CMV-	Discovery	TRUE	TRUE	TRUE	TRUE	FALSE	FALSE	FALSE	FALSE	FALSE	FALSE	FALSE
53	Female	Melanoma	Palliative	cICB	CMV-	Discovery	TRUE	TRUE	TRUE	FALSE	TRUE	TRUE	FALSE	TRUE	FALSE	FALSE	FALSE
74	Male	Mesothelic	Palliative	cICB	CMV-	Discovery	TRUE	TRUE	FALSE	FALSE	TRUE	FALSE	FALSE	FALSE	FALSE	FALSE	FALSE
90	Male	cSCC	Palliative	sICB	High Titre	Validation	TRUE	TRUE	FALSE	FALSE	TRUE	FALSE	FALSE	FALSE	TRUE	FALSE	FALSE
83	Male	Mesothelic	Palliative	cICB	Low Titre	Validation	TRUE	TRUE	TRUE	TRUE	TRUE	FALSE	FALSE	FALSE	TRUE	FALSE	FALSE
57	Female	Melanoma	Palliative	cICB	CMV-	Discovery	TRUE	TRUE	TRUE	TRUE	FALSE	TRUE	FALSE	TRUE	FALSE	FALSE	FALSE
76	Male	Melanoma	Palliative	cICB	CMV-	Validation	TRUE	TRUE	TRUE	TRUE	TRUE	FALSE	FALSE	FALSE	FALSE	FALSE	FALSE
80	Female	Melanoma	Palliative	cICB	Low Titre	Discovery	TRUE	TRUE	TRUE	TRUE	FALSE	TRUE	FALSE	TRUE	FALSE	FALSE	FALSE
79	Female	Melanoma	Palliative	cICB	Low Titre	Discovery	TRUE	TRUE	TRUE	TRUE	TRUE	TRUE	FALSE	FALSE	TRUE	FALSE	FALSE
58	Male	Melanoma	Adjuvant	sICB	CMV-	Validation	TRUE	FALSE	TRUE	TRUE	TRUE	TRUE	FALSE	FALSE	FALSE	FALSE	FALSE
56	Male	Melanoma	Adjuvant	sICB	Low Titre	Discovery	TRUE	FALSE	TRUE	TRUE	FALSE	TRUE	TRUE	FALSE	FALSE	FALSE	FALSE
51	Female	Melanoma	Palliative	cICB	Low Titre	Validation	TRUE	TRUE	TRUE	TRUE	FALSE	TRUE	TRUE	FALSE	TRUE	FALSE	FALSE
75	Female	Melanoma	Palliative	cICB	CMV-	Discovery	TRUE	TRUE	TRUE	TRUE	FALSE	TRUE	TRUE	FALSE	FALSE	FALSE	FALSE
55	Female	Melanoma	Adjuvant	sICB	CMV-	Validation	TRUE	FALSE	TRUE	TRUE	TRUE	TRUE	FALSE	FALSE	FALSE	FALSE	FALSE
70	Male	Melanoma	Palliative	cICB	High Titre	Validation	TRUE	TRUE	TRUE	TRUE	FALSE	FALSE	FALSE	FALSE	FALSE	FALSE	FALSE
80	Male	Melanoma	Palliative	sICB	CMV-	Validation	TRUE	TRUE	TRUE	TRUE	FALSE	TRUE	TRUE	FALSE	FALSE	FALSE	FALSE
62	Female	Melanoma	Adjuvant	sICB	High Titre	Discovery	TRUE	FALSE	TRUE	TRUE	FALSE	FALSE	FALSE	FALSE	TRUE	FALSE	FALSE
88	Male	Mesothelic	Palliative	cICB	CMV-	Validation	TRUE	TRUE	TRUE	TRUE	TRUE	FALSE	FALSE	FALSE	FALSE	FALSE	FALSE

62	Male	Melanoma	Palliative	cICB	CMV-	Discovery	TRUE	TRUE	TRUE	TRUE	FALSE	TRUE	FALSE	FALSE	FALSE	FALSE	FALSE
79	Female	Mesothelic	Palliative	cICB	CMV-	Discovery	TRUE	TRUE	TRUE	TRUE	FALSE	FALSE	FALSE	FALSE	FALSE	FALSE	FALSE
67	Female	Melanoma	Palliative	cICB	CMV-	Discovery	TRUE	TRUE	FALSE	FALSE	FALSE	FALSE	FALSE	FALSE	FALSE	FALSE	FALSE
39	Male	Melanoma	Palliative	cICB	CMV-	Discovery	TRUE	TRUE	TRUE	FALSE	FALSE	TRUE	TRUE	FALSE	FALSE	FALSE	FALSE
53	Male	RCC	Palliative	cICB	High Titre	Discovery	TRUE	TRUE	FALSE	FALSE	TRUE	TRUE	FALSE	FALSE	TRUE	FALSE	FALSE
68	Male	Mesothelic	Palliative	cICB	Low Titre	Validation	TRUE	TRUE	TRUE	TRUE	FALSE	FALSE	FALSE	FALSE	FALSE	FALSE	FALSE
72	Male	Melanoma	Palliative	cICB	CMV-	Discovery	TRUE	TRUE	FALSE	FALSE	TRUE	FALSE	FALSE	FALSE	FALSE	FALSE	FALSE
73	Male	Melanoma	Palliative	cICB	Low Titre	Discovery	TRUE	TRUE	TRUE	TRUE	FALSE	TRUE	TRUE	FALSE	FALSE	FALSE	FALSE
72	Male	Melanoma	Palliative	cICB	CMV-	Discovery	TRUE	TRUE	TRUE	TRUE	FALSE	TRUE	TRUE	FALSE	FALSE	FALSE	FALSE
74	Male	Melanoma	Palliative	cICB	CMV-	Discovery	TRUE	TRUE	TRUE	TRUE	FALSE	TRUE	FALSE	FALSE	FALSE	FALSE	FALSE
77	Female	RCC	Adjuvant	sICB	CMV-	Validation	TRUE	FALSE	TRUE	TRUE	TRUE	FALSE	FALSE	FALSE	FALSE	FALSE	FALSE
41	Male	RCC	Adjuvant	sICB	CMV-	Discovery	TRUE	FALSE	FALSE	FALSE	FALSE	FALSE	FALSE	FALSE	FALSE	FALSE	FALSE
88	Male	Melanoma	Palliative	sICB	CMV-	Discovery	TRUE	TRUE	TRUE	TRUE	FALSE	FALSE	FALSE	FALSE	FALSE	FALSE	FALSE
84	Male	Melanoma	Palliative	sICB	High Titre	Validation	TRUE	TRUE	TRUE	TRUE	FALSE	FALSE	FALSE	FALSE	FALSE	FALSE	FALSE
64	Male	Melanoma	Adjuvant	sICB	Low Titre	Validation	TRUE	FALSE	TRUE	TRUE	TRUE	FALSE	FALSE	FALSE	FALSE	FALSE	FALSE
86	Female	Melanoma	Palliative	sICB	High Titre	Discovery	TRUE	TRUE	FALSE	FALSE	TRUE	TRUE	FALSE	FALSE	FALSE	FALSE	FALSE
64	Female	Melanoma	Palliative	cICB	Low Titre	Validation	TRUE	TRUE	TRUE	FALSE	TRUE	FALSE	FALSE	FALSE	FALSE	FALSE	FALSE
83	Female	Melanoma	Palliative	cICB	CMV-	Validation	TRUE	TRUE	FALSE	FALSE	TRUE	FALSE	FALSE	FALSE	FALSE	FALSE	FALSE
75	Male	Melanoma	Palliative	cICB	CMV-	Discovery	TRUE	TRUE	TRUE	TRUE	FALSE	FALSE	FALSE	FALSE	FALSE	FALSE	FALSE
53	Male	RCC	Adjuvant	sICB	CMV-	Discovery	TRUE	FALSE	FALSE	FALSE	TRUE	FALSE	FALSE	FALSE	FALSE	FALSE	FALSE
77	Male	RCC	Palliative	cICB	CMV-	Discovery	TRUE	TRUE	FALSE	FALSE	TRUE	FALSE	FALSE	FALSE	FALSE	FALSE	FALSE
77	Male	Mesothelic	Palliative	cICB	Low Titre	Validation	TRUE	TRUE	TRUE	TRUE	TRUE	FALSE	FALSE	FALSE	TRUE	FALSE	FALSE
73	Male	RCC	Palliative	cICB	CMV-	Validation	TRUE	TRUE	TRUE	TRUE	TRUE	FALSE	FALSE	FALSE	FALSE	FALSE	FALSE
49	Male	Melanoma	Palliative	cICB	CMV-	Discovery	TRUE	TRUE	TRUE	TRUE	TRUE	FALSE	FALSE	FALSE	FALSE	FALSE	FALSE
86	Male	cSCC	Palliative	sICB	High Titre	Discovery	TRUE	TRUE	TRUE	TRUE	FALSE	TRUE	TRUE	FALSE	FALSE	FALSE	FALSE
77	Male	Melanoma	Adjuvant	sICB	Low Titre	Discovery	TRUE	FALSE	TRUE	TRUE	TRUE	FALSE	FALSE	FALSE	FALSE	FALSE	FALSE
85	Female	Mesothelic	Palliative	cICB	Low Titre	Validation	TRUE	TRUE	TRUE	TRUE	TRUE	FALSE	FALSE	FALSE	FALSE	FALSE	FALSE
79	Male	cSCC	Palliative	sICB	CMV-	Discovery	TRUE	TRUE	FALSE	FALSE	TRUE	FALSE	FALSE	FALSE	FALSE	FALSE	FALSE
77	Female	Melanoma	Adjuvant	sICB	Low Titre	Discovery	TRUE	FALSE	TRUE	TRUE	FALSE	FALSE	FALSE	FALSE	FALSE	FALSE	FALSE
47	Female	Mesothelic	Palliative	cICB	CMV-	Discovery	TRUE	TRUE	TRUE	TRUE	TRUE	FALSE	FALSE	FALSE	FALSE	FALSE	FALSE

66	Male	Melanoma	Adjuvant	sICB	CMV-	Validation	TRUE	FALSE	TRUE	TRUE	TRUE	TRUE	FALSE	FALSE	FALSE	FALSE	FALSE
62	Male	Melanoma	Adjuvant	sICB	CMV-	Validation	TRUE	FALSE	FALSE	FALSE	FALSE	FALSE	FALSE	FALSE	FALSE	FALSE	FALSE
64	Male	Melanoma	Adjuvant	sICB	CMV-	Discovery	TRUE	FALSE	TRUE	TRUE	TRUE	TRUE	FALSE	FALSE	FALSE	FALSE	FALSE
56	Female	Melanoma	Adjuvant	sICB	CMV-	Validation	TRUE	FALSE	TRUE	TRUE	FALSE	FALSE	FALSE	FALSE	FALSE	FALSE	FALSE
31	Female	Melanoma	Adjuvant	sICB	Low Titre	Discovery	TRUE	FALSE	TRUE	TRUE	TRUE	TRUE	FALSE	FALSE	FALSE	FALSE	FALSE
78	Male	Mesothelic	Palliative	cICB	CMV-	Validation	TRUE	TRUE	TRUE	TRUE	TRUE	FALSE	FALSE	FALSE	FALSE	FALSE	FALSE
66	Male	Melanoma	Adjuvant	sICB	CMV-	Discovery	TRUE	FALSE	TRUE	TRUE	TRUE	FALSE	FALSE	FALSE	FALSE	FALSE	FALSE
78	Male	Melanoma	Palliative	sICB	CMV-	Discovery	TRUE	TRUE	FALSE	FALSE	TRUE	FALSE	FALSE	FALSE	FALSE	FALSE	FALSE
94	Female	Melanoma	Palliative	sICB	High Titre	Validation	TRUE	TRUE	FALSE	FALSE	FALSE	FALSE	FALSE	FALSE	FALSE	FALSE	FALSE
51	Female	Melanoma	Palliative	cICB	CMV-	Discovery	TRUE	TRUE	TRUE	TRUE	FALSE	FALSE	FALSE	FALSE	FALSE	FALSE	FALSE
68	Female	Melanoma	Adjuvant	sICB	CMV-	Discovery	TRUE	FALSE	FALSE	FALSE	FALSE	FALSE	FALSE	FALSE	FALSE	FALSE	FALSE
66	Female	Melanoma	Palliative	cICB	CMV-	Validation	TRUE	TRUE	FALSE	FALSE	FALSE	TRUE	FALSE	FALSE	FALSE	FALSE	FALSE
80	Female	Mesothelic	Palliative	cICB	Low Titre	Discovery	TRUE	TRUE	TRUE	TRUE	FALSE	FALSE	FALSE	FALSE	FALSE	FALSE	FALSE
71	Male	Melanoma	Palliative	cICB	CMV-	Discovery	TRUE	TRUE	TRUE	TRUE	FALSE	FALSE	FALSE	FALSE	FALSE	FALSE	FALSE
63	Male	Melanoma	Palliative	cICB	CMV-	Validation	TRUE	TRUE	TRUE	TRUE	FALSE	TRUE	FALSE	FALSE	FALSE	FALSE	FALSE
72	Female	Melanoma	Palliative	cICB	CMV-	Discovery	TRUE	TRUE	TRUE	TRUE	FALSE	FALSE	FALSE	FALSE	FALSE	FALSE	FALSE
67	Female	Colon	Palliative	sICB	High Titre	Discovery	TRUE	TRUE	TRUE	TRUE	FALSE	FALSE	FALSE	FALSE	FALSE	FALSE	FALSE
74	Female	Melanoma	Palliative	cICB	Low Titre	Validation	TRUE	TRUE	TRUE	TRUE	FALSE	TRUE	FALSE	FALSE	FALSE	FALSE	FALSE
69	Male	Melanoma	Palliative	cICB	High Titre	Discovery	TRUE	TRUE	TRUE	TRUE	FALSE	TRUE	FALSE	FALSE	FALSE	FALSE	FALSE
67	Male	Melanoma	Palliative	cICB	Low Titre	Validation	TRUE	TRUE	TRUE	TRUE	FALSE	FALSE	FALSE	FALSE	FALSE	FALSE	FALSE
88	Male	Melanoma	Palliative	sICB	CMV-	Discovery	TRUE	TRUE	TRUE	TRUE	FALSE	FALSE	FALSE	FALSE	FALSE	FALSE	FALSE
82	Male	Melanoma	Palliative	cICB	CMV-	Discovery	TRUE	TRUE	TRUE	TRUE	FALSE	TRUE	FALSE	FALSE	FALSE	FALSE	FALSE
85	Male	Melanoma	Palliative	sICB	CMV-	Discovery	TRUE	TRUE	TRUE	TRUE	FALSE	FALSE	FALSE	FALSE	FALSE	FALSE	FALSE
60	Male	Melanoma	Palliative	cICB	Low Titre	Discovery	TRUE	TRUE	TRUE	TRUE	FALSE	TRUE	TRUE	FALSE	FALSE	FALSE	FALSE
86	Male	Mesothelic	Palliative	cICB	CMV-	Discovery	TRUE	TRUE	TRUE	TRUE	FALSE	FALSE	FALSE	FALSE	FALSE	FALSE	FALSE
80	Male	Melanoma	Palliative	cICB	CMV-	Discovery	TRUE	TRUE	TRUE	TRUE	FALSE	FALSE	FALSE	FALSE	FALSE	FALSE	FALSE
67	Female	RCC	Palliative	cICB	High Titre	Validation	TRUE	TRUE	TRUE	TRUE	FALSE	TRUE	FALSE	FALSE	FALSE	FALSE	FALSE
59	Female	Melanoma	Adjuvant	sICB	High Titre	Discovery	TRUE	FALSE	FALSE	FALSE	FALSE	FALSE	FALSE	FALSE	FALSE	FALSE	FALSE
76	Female	RCC	Palliative	cICB	High Titre	Discovery	TRUE	TRUE	TRUE	TRUE	FALSE	FALSE	FALSE	FALSE	FALSE	FALSE	FALSE
73	Male	Melanoma	Palliative	cICB	High Titre	Validation	TRUE	TRUE	TRUE	TRUE	FALSE	TRUE	TRUE	FALSE	FALSE	FALSE	FALSE

56	Male	Melanoma	Palliative	cICB	CMV-	Discovery	TRUE	TRUE	TRUE	TRUE	FALSE	FALSE	FALSE	FALSE	FALSE	FALSE	FALSE
60	Male	Melanoma	Palliative	cICB	CMV-	Validation	TRUE	TRUE	TRUE	TRUE	FALSE	FALSE	FALSE	FALSE	FALSE	FALSE	FALSE
77	Male	Melanoma	Adjuvant	sICB	CMV-	Discovery	TRUE	FALSE	TRUE	TRUE	FALSE	FALSE	FALSE	FALSE	FALSE	FALSE	FALSE
68	Male	Melanoma	Adjuvant	sICB	CMV-	Validation	TRUE	FALSE	FALSE	FALSE	FALSE	FALSE	FALSE	FALSE	FALSE	FALSE	FALSE
76	Male	Mesothelic	Palliative	cICB	CMV-	Validation	TRUE	TRUE	TRUE	TRUE	FALSE	FALSE	FALSE	FALSE	FALSE	FALSE	FALSE
60	Male	Melanoma	Palliative	cICB	CMV-	Validation	TRUE	TRUE	TRUE	TRUE	FALSE	TRUE	FALSE	FALSE	FALSE	FALSE	FALSE
69	Female	RCC	Adjuvant	sICB	High Titre	Validation	TRUE	FALSE	TRUE	TRUE	FALSE	FALSE	FALSE	FALSE	FALSE	FALSE	FALSE
72	Male	Melanoma	Adjuvant	sICB	CMV-	Validation	TRUE	FALSE	TRUE	TRUE	FALSE	FALSE	FALSE	FALSE	FALSE	FALSE	FALSE
77	Male	Melanoma	Palliative	sICB	CMV-	Validation	TRUE	TRUE	FALSE	FALSE	FALSE	FALSE	FALSE	FALSE	FALSE	FALSE	FALSE
73	Male	Melanoma	Palliative	cICB	CMV-	Discovery	TRUE	TRUE	TRUE	TRUE	FALSE	TRUE	FALSE	FALSE	FALSE	FALSE	FALSE
88	Female	cSCC	Palliative	sICB	Low Titre	Discovery	TRUE	TRUE	FALSE	FALSE	FALSE	FALSE	FALSE	FALSE	FALSE	FALSE	FALSE
42	Female	Mesothelic	Palliative	cICB	High Titre	Validation	TRUE	TRUE	FALSE	FALSE	FALSE	FALSE	FALSE	FALSE	FALSE	FALSE	FALSE
44	Female	Melanoma	Palliative	cICB	High Titre	Validation	TRUE	TRUE	TRUE	TRUE	FALSE	FALSE	FALSE	FALSE	FALSE	FALSE	FALSE
72	Male	Melanoma	Palliative	cICB	CMV-	Validation	TRUE	TRUE	FALSE	FALSE	FALSE	FALSE	FALSE	FALSE	FALSE	FALSE	FALSE
58	Female	RCC	Adjuvant	sICB	CMV-	Discovery	TRUE	FALSE	TRUE	TRUE	FALSE	FALSE	FALSE	FALSE	FALSE	FALSE	FALSE
85	Male	cSCC	Palliative	sICB	CMV-	Validation	TRUE	TRUE	FALSE	FALSE	FALSE	FALSE	FALSE	FALSE	FALSE	FALSE	FALSE
79	Male	Mesothelic	Palliative	cICB	CMV-	Discovery	TRUE	TRUE	FALSE	FALSE	FALSE	FALSE	FALSE	FALSE	FALSE	FALSE	FALSE
72	Male	Mesothelic	Palliative	cICB	CMV-	Discovery	TRUE	TRUE	FALSE	FALSE	FALSE	FALSE	FALSE	FALSE	FALSE	FALSE	FALSE
52	Male	Melanoma	Palliative	cICB	CMV-	Discovery	TRUE	TRUE	TRUE	TRUE	FALSE	FALSE	FALSE	FALSE	FALSE	FALSE	FALSE
76	Male	Colon	Palliative	cICB	CMV-	Validation	TRUE	TRUE	FALSE	FALSE	FALSE	FALSE	FALSE	FALSE	FALSE	FALSE	FALSE
69	Male	Melanoma	Adjuvant	sICB	CMV-	Validation	TRUE	FALSE	TRUE	TRUE	FALSE	FALSE	FALSE	FALSE	FALSE	FALSE	FALSE
80	Female	Melanoma	Adjuvant	sICB	High Titre	Validation	TRUE	FALSE	TRUE	TRUE	FALSE	FALSE	FALSE	FALSE	FALSE	FALSE	FALSE
85	Female	Melanoma	Palliative	cICB	CMV-	Discovery	TRUE	TRUE	TRUE	TRUE	FALSE	FALSE	FALSE	FALSE	FALSE	FALSE	FALSE
65	Male	Melanoma	Palliative	cICB	High Titre	Validation	TRUE	TRUE	TRUE	TRUE	FALSE	FALSE	FALSE	FALSE	FALSE	FALSE	FALSE
62	Male	Melanoma	Adjuvant	sICB	Low Titre	Validation	TRUE	FALSE	FALSE	FALSE	FALSE	FALSE	FALSE	FALSE	FALSE	FALSE	FALSE
84	Female	Melanoma	Palliative	sICB	High Titre	Discovery	TRUE	TRUE	FALSE	FALSE	FALSE	FALSE	FALSE	FALSE	FALSE	FALSE	FALSE
48	Male	Melanoma	Palliative	cICB	CMV-	Validation	TRUE	TRUE	TRUE	TRUE	FALSE	FALSE	FALSE	FALSE	FALSE	FALSE	FALSE
54	Female	Melanoma	Palliative	cICB	Low Titre	Discovery	TRUE	TRUE	TRUE	TRUE	FALSE	FALSE	FALSE	FALSE	FALSE	FALSE	FALSE
74	Male	Mesothelic	Palliative	cICB	Low Titre	Validation	TRUE	TRUE	FALSE	FALSE	FALSE	FALSE	FALSE	FALSE	FALSE	FALSE	FALSE
73	Male	Melanoma	Adjuvant	sICB	CMV-	Discovery	TRUE	FALSE	TRUE	TRUE	FALSE	FALSE	FALSE	FALSE	FALSE	FALSE	FALSE

69	Female	Melanoma	Adjuvant	sICB	High Titre	Validation	TRUE	FALSE	FALSE	FALSE	FALSE	FALSE	FALSE	FALSE	FALSE	FALSE	FALSE
87	Male	cSCC	Palliative	sICB	Low Titre	Validation	TRUE	TRUE	FALSE	FALSE	FALSE	FALSE	FALSE	FALSE	FALSE	FALSE	FALSE
77	Male	Mesothelic	Palliative	cICB	High Titre	Validation	TRUE	TRUE	FALSE	FALSE	FALSE	FALSE	FALSE	FALSE	FALSE	FALSE	FALSE
74	Female	Melanoma	Palliative	cICB	Low Titre	Validation	TRUE	TRUE	TRUE	TRUE	FALSE	FALSE	FALSE	FALSE	FALSE	FALSE	FALSE
42	Female	Colon	Palliative	sICB	Low Titre	Validation	TRUE	TRUE	FALSE	FALSE	FALSE	FALSE	FALSE	FALSE	FALSE	FALSE	FALSE
57	Female	Melanoma	Adjuvant	sICB	CMV-	Discovery	TRUE	FALSE	TRUE	TRUE	FALSE	FALSE	FALSE	FALSE	FALSE	FALSE	FALSE
75	Male	Colon	Palliative	sICB	High Titre	Discovery	TRUE	TRUE	FALSE	FALSE	FALSE	FALSE	FALSE	FALSE	FALSE	FALSE	FALSE
81	Male	cSCC	Palliative	sICB	CMV-	Validation	TRUE	TRUE	FALSE	FALSE	FALSE	FALSE	FALSE	FALSE	FALSE	FALSE	FALSE
70	Male	Melanoma	Palliative	cICB	CMV-	Validation	TRUE	TRUE	TRUE	TRUE	FALSE	FALSE	FALSE	FALSE	FALSE	FALSE	FALSE
70	Male	Melanoma	Palliative	cICB	High Titre	Discovery	TRUE	TRUE	TRUE	TRUE	FALSE	FALSE	FALSE	FALSE	FALSE	FALSE	FALSE
68	Male	Melanoma	Palliative	cICB	High Titre	Validation	TRUE	TRUE	TRUE	TRUE	FALSE	FALSE	FALSE	FALSE	FALSE	FALSE	FALSE
74	Female	Melanoma	Palliative	cICB	High Titre	Validation	TRUE	TRUE	TRUE	TRUE	FALSE	FALSE	FALSE	FALSE	FALSE	FALSE	FALSE
77	Male	Mesothelic	Palliative	cICB	CMV-	Validation	TRUE	TRUE	FALSE	FALSE	FALSE	FALSE	FALSE	FALSE	FALSE	FALSE	FALSE
60	Female	Mesothelic	Palliative	cICB	CMV-	Validation	TRUE	TRUE	FALSE	FALSE	FALSE	FALSE	FALSE	FALSE	FALSE	FALSE	FALSE
72	Male	Melanoma	Palliative	cICB	CMV-	Discovery	TRUE	TRUE	FALSE	FALSE	FALSE	FALSE	FALSE	FALSE	FALSE	FALSE	FALSE
75	Female	Melanoma	Palliative	sICB	CMV-	Discovery	TRUE	TRUE	TRUE	TRUE	FALSE	FALSE	FALSE	FALSE	FALSE	FALSE	FALSE
74	Female	Melanoma	Adjuvant	sICB	CMV-	Validation	TRUE	FALSE	TRUE	TRUE	FALSE	FALSE	FALSE	FALSE	FALSE	FALSE	FALSE
65	Male	Mesothelic	Palliative	cICB	CMV-	Discovery	TRUE	TRUE	FALSE	FALSE	FALSE	FALSE	FALSE	FALSE	FALSE	FALSE	FALSE
70	Male	Melanoma	Palliative	cICB	CMV-	Validation	TRUE	TRUE	FALSE	FALSE	FALSE	FALSE	FALSE	FALSE	FALSE	FALSE	FALSE
60	Male	Colon	Palliative	cICB	CMV-	Validation	TRUE	TRUE	FALSE	FALSE	FALSE	FALSE	FALSE	FALSE	FALSE	FALSE	FALSE
74	Male	Melanoma	Palliative	sICB	High Titre	Discovery	TRUE	TRUE	TRUE	TRUE	FALSE	FALSE	FALSE	FALSE	FALSE	FALSE	FALSE
59	Female	Melanoma	Palliative	cICB	Low Titre	Validation	TRUE	TRUE	TRUE	TRUE	FALSE	FALSE	FALSE	FALSE	FALSE	FALSE	FALSE
85	Female	Melanoma	Palliative	sICB	CMV-	Validation	TRUE	TRUE	FALSE	FALSE	FALSE	FALSE	FALSE	FALSE	FALSE	FALSE	FALSE
42	Male	Melanoma	Palliative	cICB	Low Titre	Discovery	TRUE	TRUE	FALSE	FALSE	FALSE	FALSE	FALSE	FALSE	FALSE	FALSE	FALSE
73	Female	Melanoma	Palliative	cICB	CMV-	Validation	TRUE	TRUE	FALSE	FALSE	FALSE	FALSE	FALSE	FALSE	FALSE	FALSE	FALSE
82	Male	Melanoma	Palliative	sICB	CMV-	Validation	TRUE	TRUE	FALSE	FALSE	FALSE	FALSE	FALSE	FALSE	FALSE	FALSE	FALSE
78	Male	Mesothelic	Palliative	cICB	CMV-	Discovery	TRUE	TRUE	FALSE	FALSE	FALSE	FALSE	FALSE	FALSE	FALSE	FALSE	FALSE
55	Male	Melanoma	Adjuvant	sICB	CMV-	Discovery	TRUE	FALSE	FALSE	FALSE	FALSE	FALSE	FALSE	FALSE	FALSE	FALSE	FALSE
62	Male	Melanoma	Adjuvant	sICB	CMV-	Validation	TRUE	FALSE	FALSE	FALSE	FALSE	FALSE	FALSE	FALSE	FALSE	FALSE	FALSE
57	Female	Melanoma	Adjuvant	sICB	High Titre	Validation	TRUE	FALSE	FALSE	FALSE	FALSE	FALSE	FALSE	FALSE	FALSE	FALSE	FALSE

70	Female	Colon	Palliative	cICB	High Titre	Discovery	TRUE	TRUE	FALSE	FALSE	FALSE	FALSE	FALSE	FALSE	FALSE	FALSE	FALSE
62	Male	Melanoma	Palliative	cICB	CMV-	Validation	TRUE	TRUE	FALSE	FALSE	FALSE	FALSE	FALSE	FALSE	FALSE	FALSE	FALSE
69	Male	Melanoma	Palliative	cICB	CMV-	Validation	TRUE	TRUE	FALSE	FALSE	FALSE	FALSE	FALSE	FALSE	FALSE	FALSE	FALSE
45	Female	Melanoma	Palliative	cICB	CMV-	Validation	TRUE	TRUE	FALSE	FALSE	FALSE	FALSE	FALSE	FALSE	FALSE	FALSE	FALSE
76	Male	Melanoma	Palliative	cICB	CMV-	Validation	TRUE	TRUE	FALSE	FALSE	FALSE	FALSE	FALSE	FALSE	FALSE	FALSE	FALSE
85	Male	cSCC	Palliative	sICB	CMV-	Discovery	TRUE	TRUE	FALSE	FALSE	FALSE	FALSE	FALSE	FALSE	FALSE	FALSE	FALSE
60	Male	Melanoma	Palliative	cICB	CMV-	Discovery	TRUE	TRUE	FALSE	FALSE	FALSE	FALSE	FALSE	FALSE	FALSE	FALSE	FALSE
79	Male	Melanoma	Adjuvant	sICB	CMV-	Validation	TRUE	FALSE	FALSE	FALSE	FALSE	FALSE	FALSE	FALSE	FALSE	FALSE	FALSE
61	Female	Melanoma	Adjuvant	sICB	CMV-	Validation	TRUE	FALSE	FALSE	FALSE	FALSE	FALSE	FALSE	FALSE	FALSE	FALSE	FALSE
75	Male	Melanoma	Palliative	cICB	CMV-	Discovery	TRUE	TRUE	FALSE	FALSE	FALSE	FALSE	FALSE	FALSE	FALSE	FALSE	FALSE
66	Male	Colon	Palliative	cICB	High Titre	Validation	TRUE	TRUE	FALSE	FALSE	FALSE	FALSE	FALSE	FALSE	FALSE	FALSE	FALSE
77	Female	Melanoma	Palliative	sICB	High Titre	Validation	TRUE	TRUE	FALSE	FALSE	FALSE	FALSE	FALSE	FALSE	FALSE	FALSE	FALSE
87	Female	Melanoma	Palliative	sICB	High Titre	Validation	TRUE	TRUE	FALSE	FALSE	FALSE	FALSE	FALSE	FALSE	FALSE	FALSE	FALSE
73	Male	cSCC	Palliative	sICB	Low Titre	Validation	TRUE	TRUE	FALSE	FALSE	FALSE	FALSE	FALSE	FALSE	FALSE	FALSE	FALSE
68	Male	Melanoma	Palliative	cICB	CMV-	Discovery	TRUE	TRUE	FALSE	FALSE	FALSE	FALSE	FALSE	FALSE	FALSE	FALSE	FALSE
47	Female	Melanoma	Palliative	cICB	High Titre	Discovery	TRUE	TRUE	FALSE	FALSE	FALSE	FALSE	FALSE	FALSE	FALSE	FALSE	FALSE
69	Female	RCC	Adjuvant	sICB	CMV-	Discovery	TRUE	FALSE	FALSE	FALSE	FALSE	FALSE	FALSE	FALSE	FALSE	FALSE	FALSE
49	Female	Melanoma	Palliative	cICB	CMV-	Validation	TRUE	TRUE	FALSE	FALSE	FALSE	FALSE	FALSE	FALSE	FALSE	FALSE	FALSE
79	Male	cSCC	Palliative	sICB	CMV-	Validation	TRUE	TRUE	FALSE	FALSE	FALSE	FALSE	FALSE	FALSE	FALSE	FALSE	FALSE
68	Male	Melanoma	Palliative	cICB	High Titre	Validation	TRUE	TRUE	FALSE	FALSE	FALSE	FALSE	FALSE	FALSE	FALSE	FALSE	FALSE
68	Female	Colon	Palliative	sICB	Low Titre	Discovery	TRUE	TRUE	FALSE	FALSE	FALSE	FALSE	FALSE	FALSE	FALSE	FALSE	FALSE
78	Male	Mesothelic	Palliative	cICB	High Titre	Discovery	TRUE	TRUE	FALSE	FALSE	FALSE	FALSE	FALSE	FALSE	FALSE	FALSE	FALSE
74	Male	Melanoma	Palliative	cICB	High Titre	Discovery	TRUE	TRUE	FALSE	FALSE	FALSE	FALSE	FALSE	FALSE	FALSE	FALSE	FALSE
35	Male	Melanoma	Palliative	cICB	Low Titre	Validation	TRUE	TRUE	FALSE	FALSE	FALSE	FALSE	FALSE	FALSE	FALSE	FALSE	FALSE
58	Female	Melanoma	Adjuvant	sICB	High Titre	Validation	TRUE	FALSE	FALSE	FALSE	FALSE	FALSE	FALSE	FALSE	FALSE	FALSE	FALSE

Supplementary Table 5- Cohort missingness table

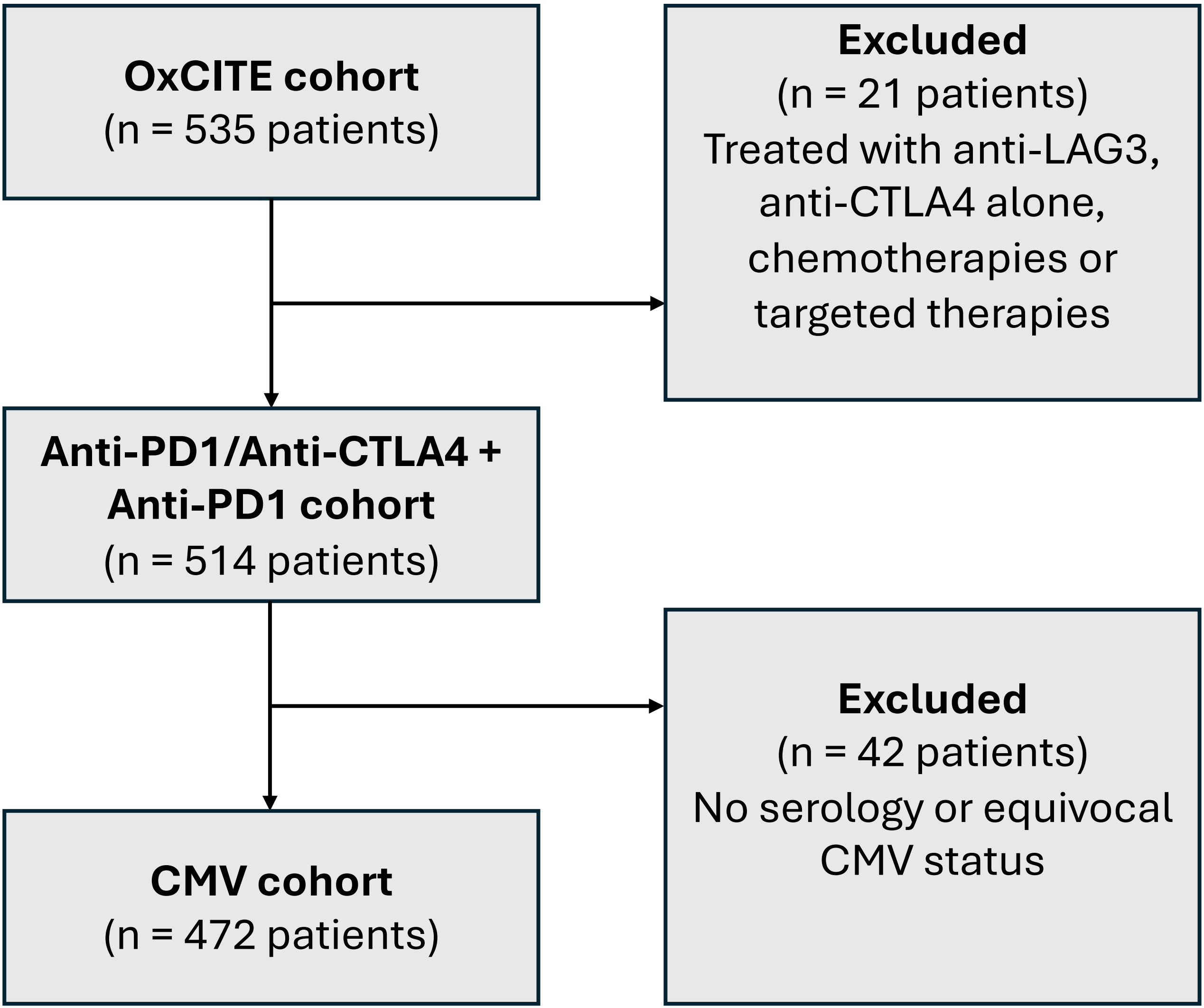

Supplementary Figure 1. Exclusion criteria flow chart

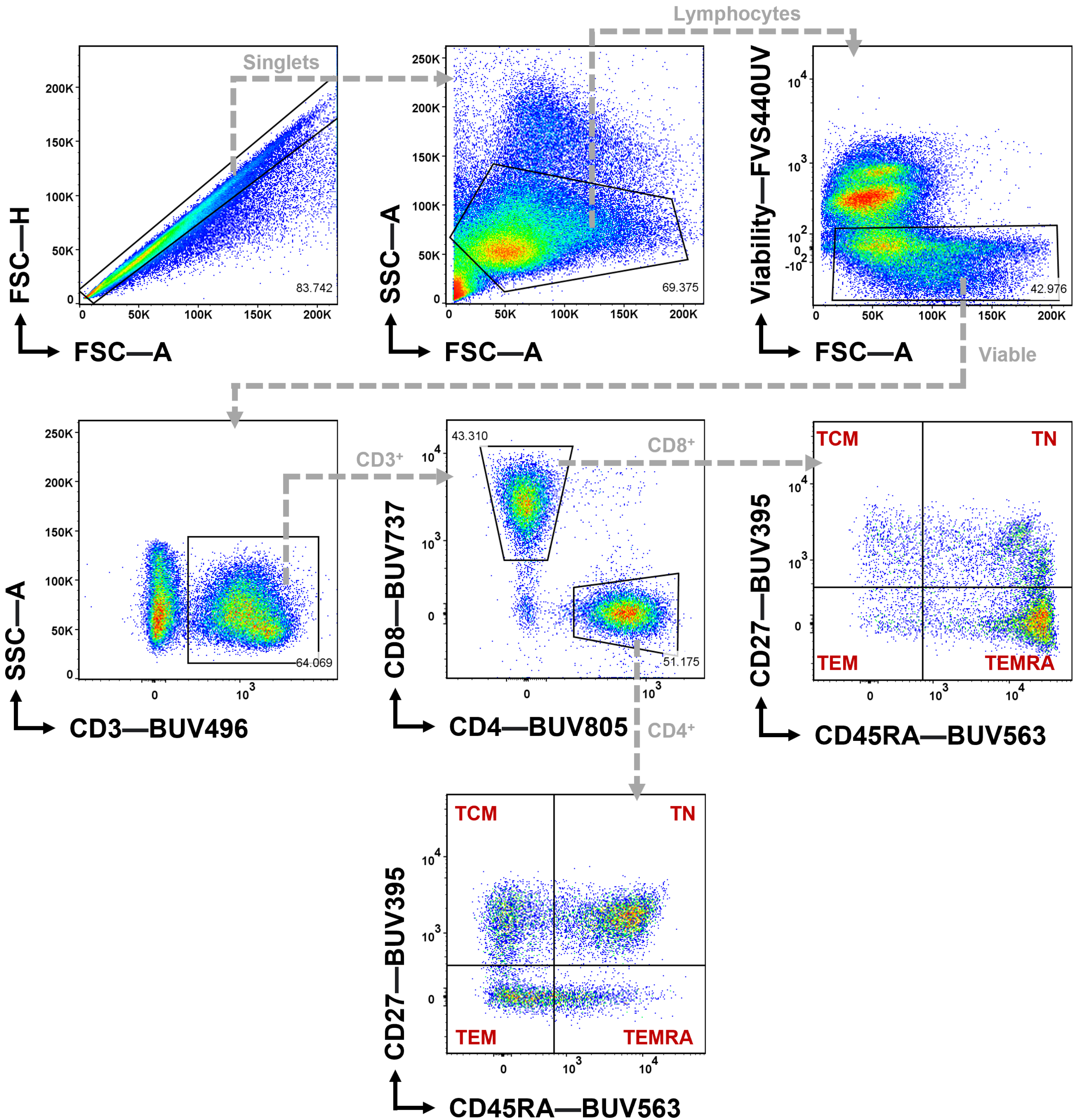

Supplementary Figure 2. Phenotyping gating strategy
